# The contribution of tropical long-term studies to mycology

**DOI:** 10.1186/s43008-024-00166-5

**Published:** 2024-11-11

**Authors:** Jeffery K. Stallman, Danny Haelewaters, Rachel A. Koch Bach, Mia Brann, Samira Fatemi, Paula Gomez-Zapata, Dillon R. Husbands, Blaise Jumbam, Patricia J. Kaishian, Ariana Moffitt, M. Catherine Aime

**Affiliations:** 1 Department of Botany and Plant Pathology, Purdue University, West Lafayette, IN 47901 USA; 2 Department of Ecology and Evolutionary Biology, University of Colorado Boulder, Boulder, CO 80309 USA; 3 Biology Centre of the Czech Academy of Sciences, Institute of Entomology, 370 05 České Budějovice, Czech Republic; 4 Faculty of Science, University of South Bohemia, 370 05 České Budějovice, Czech Republic; 5 Department of Biology, Northern Arizona University, Flagstaff, AZ 86001 USA; 6 Pacific Biosciences Research Center, University of Hawaiʻi at Mānoa, Honolulu, HI 96822 USA; 7 Department of Forest Mycology and Plant Pathology, Uppsala Biocenter, Swedish University of Agricultural Sciences, 750 07 Uppsala, Sweden; 8 Department of Agriculture, University of Guyana, Turkeyen Campus, Greater Georgetown, Guyana; 9 Department of Plant Science and Landscape Architecture, University of Maryland, College Park, 20742 MD USA; 10 New York State Museum, Albany, NY 12230 USA; 11 Department of Nutrition Science, Purdue University, West Lafayette, IN 47901 USA

**Keywords:** Biodiversity, Biogeography, Citizen science, Conservation, Endemic fungi, Guiana shield, Taxonomy

## Abstract

Fungi are arguably the most diverse eukaryotic kingdom of organisms in terms of number of estimated species, trophic and life history strategies, and their functions in ecosystems. However, our knowledge of fungi is limited due to a distributional bias; the vast majority of available data on fungi have been compiled from non-tropical regions. Far less is known about fungi from tropical regions, with the bulk of these data being temporally limited surveys for fungal species diversity. Long-term studies (LTS), or repeated sampling from the same region over extended periods, are necessary to fully capture the extent of species diversity in a region, but LTS of fungi from tropical regions are almost non-existent. In this paper, we discuss the contributions of LTS of fungi in tropical regions to alpha diversity, ecological and functional diversity, biogeography, hypothesis testing, and conservation—with an emphasis on an ongoing tropical LTS in the Pakaraima Mountains of Guyana. We show how these contributions refine our understanding of Fungi. We also show that public data repositories such as NCBI, IUCN, and iNaturalist contain less information on tropical fungi compared to non-tropical fungi, and that these discrepancies are more pronounced in fungi than in plants and animals.

**Supplementary Information:**

The online version contains supplementary material available at 10.1186/s43008-024-00166-5.

## Resumen

Los hongos son, posiblemente, el reino más diverso dentro del dominio Eukaryota en cuanto al número de especies estimadas, estrategias tróficas, ciclos de vida y funciones en los ecosistemas. No obstante, nuestro conocimiento sobre ellos es limitado debido a un sesgo de distribución: la mayoría de los datos disponibles provienen de regiones no tropicales. Se sabe mucho menos sobre los hongos de las zonas tropicales, y la mayor parte de los estudios realizados en estas áreas han sido de corta duración, centrados en la diversidad de especies fúngicas. Los estudios a largo plazo (LTS, por sus siglas en inglés), es decir, muestreos repetidos en una misma región durante períodos prolongados, son fundamentales para captar de manera completa la diversidad de especies en una zona. Sin embargo, los LTS de hongos en regiones tropicales son prácticamente inexistentes. En este artículo, analizamos las contribuciones de los LTS de hongos en zonas tropicales en relación con la diversidad alfa, la diversidad ecológica y funcional, la biogeografía, la prueba de hipótesis y la conservación, destacando un estudio LTS en curso en las montañas Pakaraima de Guyana. Mostramos cómo estos estudios enriquecen nuestra comprensión de los hongos. Además, demostramos que los repositorios públicos de datos como NCBI, UICN e iNaturalist contienen menos información sobre los hongos tropicales en comparación con los de regiones no tropicales, y que estas discrepancias son más marcadas en los hongos que en las plantas y los animales.

## Introduction

Fungi are extremely diverse in terms of species richness and ecological functions. About 155,000 species are formally described (Kirk 2023) out of approximately 2.5 million (Niskanen et al. 2023), although estimates vary between 600,000 and 12,000,000 (Mora et al. 2011; Wu et al. 2019). Even though fungi fulfill essential ecological roles in our ecosystems as saprotrophs, mutualists, and parasites and pathogens (Willis 2018), our knowledge of fungal diversity lags behind that of other groups of organisms. Reasons for this include: (i) most fungi are microscopic or produce cryptic, temporal sporocarps, (ii) they often occupy highly specialized substrates or microhabitats, and (iii) potential hotspots for fungal biodiversity remain underexplored (Blackwell 2011). Traditionally, our knowledge of fungal diversity—inclusive of species richness, gene richness, and ecological and functional roles—has been based on information from the temperate Northern Hemisphere (e.g., Aime and Brearley 2012; Quandt and Haelewaters 2021).

Terrestrial tropical ecosystems include the forests, savannahs, and other habitat types that lie between the Tropic of Cancer and the Tropic of Capricorn where the latitudinal diversity gradient hypothesis posits that maximum alpha diversity is found (Pianka 1966; Hillebrand 2004). Although the warm, wet, and relatively aseasonal climate of tropical forests is favorable for maintaining potentially higher fungal diversity than anywhere else in the world, current data show this is variable based on taxonomic and functional groups. For example, increased tropical diversity has been supported for ecological groups, such as endophytes (Arnold and Lutzoni 2007), and proposed for plant-pathogenic microfungi (Shivas and Hyde 1997) and arthropod-associated *Laboulbeniales* (Weir and Hammond 1997), while ectomycorrhizal (ECM) fungi and certain classes such as *Leotiomycetes* are believed to be more diverse in non-tropical regions (Tedersoo and Nara 2010; Tedersoo et al. 2014). Evidence from meta-analyses of high-throughput sequencing (HTS) studies of soils confirms that tropical woodlands, highlands, and lowland and montane forests have some of the highest alpha diversity of fungi in the world, excluding extremely wet or arid regions (Mikryukov et al. 2023; Niskanen et al. 2023; but see Větrovský et al. 2019).

Aspects of fungal biology make documenting their diversity harder than many other organismal groups. The cryptic nature and ephemeral sporocarps of fungal species make it difficult to find and collect even a small proportion of the total number of (macrofungal) species during a single sampling effort. The factors triggering sporocarp production are multiple and often unpredictable, so to achieve a proper census, repeated sampling over many years is necessary. Additionally, high variability in species found across time and space may occur in both studies collecting sporocarps (Lodge and Cantrell 1995) and HTS of soils (Izzo et al. 2005). The lack of long-term datasets in tropical habitats has hindered the ability to estimate how well sampling efforts are capturing the full suite of fungal species richness (Aime and Brearley 2012).

### Long-term studies in the tropics

Long-term studies (LTS) involve repeated sampling of a specific field site over extended periods either in a standardized manner (i.e., plot-based or along transects at regular time intervals) or incidentally (O’Dell et al. 2004). In this paper, we focus on scientific contributions from LTS of at least one year in length. We note this is an arbitrary time frame because studies with repeated, frequent sampling < 1 year may still make significant contributions and studies > 1 year may only occur once annually for a short period of time. By and large, LTS produce datasets that offer numerous advantages compared to those resulting from single sampling events, allowing comparisons over time as populations, communities, ecosystems, and environments change.

Datasets from fungal LTS sites can include vouchered specimens and cultures with associated metadata, including but not limited to phenological, ecological and climatic measurements, Sanger sequencing, HTS, and genomic-scale molecular data. However, very few mycological studies have been designed to collect LTS data from tropical systems (Hyde et al. 2020). Locations of published examples of LTS from the tropics focusing on species inventories include Benin (Houdanon et al. 2022), Cameroon (Jumbam et al. 2019), southern China (Li et al. 2018), Colombia (Vasco-Palacios et al. 2005; López-Quintero et al. 2012), Dominican Republic (Angelini 2022), Ecuador (Læssøe and Petersen 2011; Vandegrift 2023), the Greater Antilles (Lodge 2018), Hawaiʻi, USA (Hemmes and Desjardin 2001), Honduras (Haelewaters et al. 2021b), Panama (Piepenbring et al. 2015), São Tomé and Príncipe (Desjardin and Perry 2022), and northern Thailand (Hyde et al. 2018). Studies vary in time (two years in Panama to 20 years in the Greater Antilles), structure (plot-based surveys in Cameroon to general field surveys in Hawaiʻi), and may be ongoing (Honduras) or completed (São Tomé and Príncipe), although publishing data from “completed” studies may still be occurring (e.g., Desjardin and Perry 2022). Additionally, LTS studies focusing on other aspects of fungal biology, such as the impact of nitrogen addition on fungal communities, have occurred in China (He et al. 2021) and Panama (Corrales et al. 2017). A data-rich tropical LTS comes from the Pakaraima Mountains of western Guyana within South America’s Guiana Shield. Known as the Upper Potaro River Study (UPRS) due to the location of the study site within the Upper Potaro River Basin at the base of Mount Ayanganna, this LTS has produced data from nearly 20 years of continuous sampling.

The UPRS is based on long-term plots that were established in the year 2000. Three 1-ha plots in primary monodominant forests of the ECM canopy tree *Dicymbe corymbosa* and three 1-ha plots in surrounding mixed forest were sampled for seven years during the primary rainy season (roughly May–June). Data collected include complete plot counts of sporocarps, vouchers, a plant species census in plots, daily rainfall and temperature, Sanger sequencing data from sporocarps and colonized plant root tips, and HTS data from soil, leaf litter, and ECM root tips (e.g., Henkel et al. 2012; Smith et al. 2013; Torres-Cruz 2023). Additionally, off-plot opportunistic sampling for microfungi, plant pathogens, and mushrooms was conducted over 20 years in the same region during both rainy seasons (roughly May–June and December–January) and in neighboring regions either contiguous or discontiguous with UPRS. To date, approximately 1500 species of fungi have been documented from these areas combined, of which 500 are putative new species (Blackwell 2011; Table S1). Approximately 50% of collected vouchers are DNA barcoded, allowing for comparisons and identification of sequences in HTS-generated datasets (Smith et al. 2013), and genomic data have been generated for select species. The nested sampling design allows for multiple types of comparative analyses (e.g., mixed plot vs. ECM plots, UPRS site vs. similar discontiguous sites, etc.). At the time of writing, 85 papers have been published from this LTS, of which 65 deal with alpha diversity and 33 with comparative analyses, functional or genetic diversity, or other topics (Table S2; some papers address multiple topics). Below, we draw on LTS studies from the tropics, with an emphasis on the UPRS, to highlight knowledge advancements in alpha, ecological, and functional diversity, biogeography, hypothesis testing, and conservation of Fungi.

## Contributions of long-term studies in the tropics

### Alpha diversity

#### Discovery of new species and genera

**Fig. 1:**
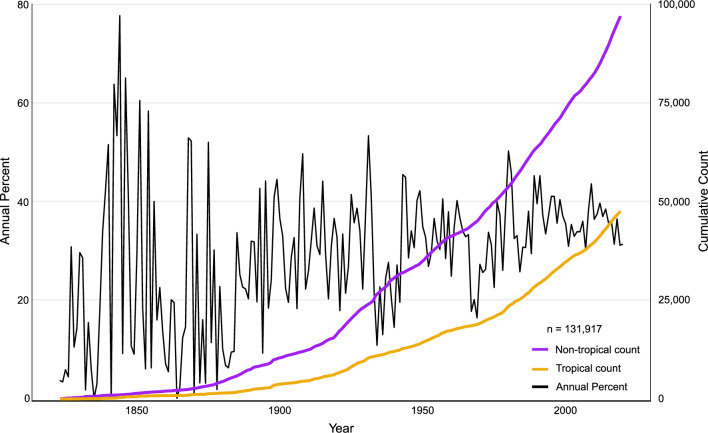
MycoBank.org data on tropical and non-tropical fungal name depositions. Cumulative counts of fungal species name depositions in Mycobank.org from tropical and non-tropical locations, and annual percentage of tropical fungal names deposited in Mycobank from 1823 to November 2020

To explore alpha diversity over time between tropical and non-tropical locations, we examined all names deposited in MycoBank (https://mycobank.org) since 1823. Of the names assignable to a region, 33% were of tropical origin and 67% were of non-tropical origin (Fig. 1, methods in Additional file 1). While the annual percentage of tropical taxa described has stabilized at 30–45% in the last 30 years, only twice within the past 100 years have more tropical than non-tropical taxa been described, in 1931 and 1980 (Fig. 1).

**Fig. 2:**
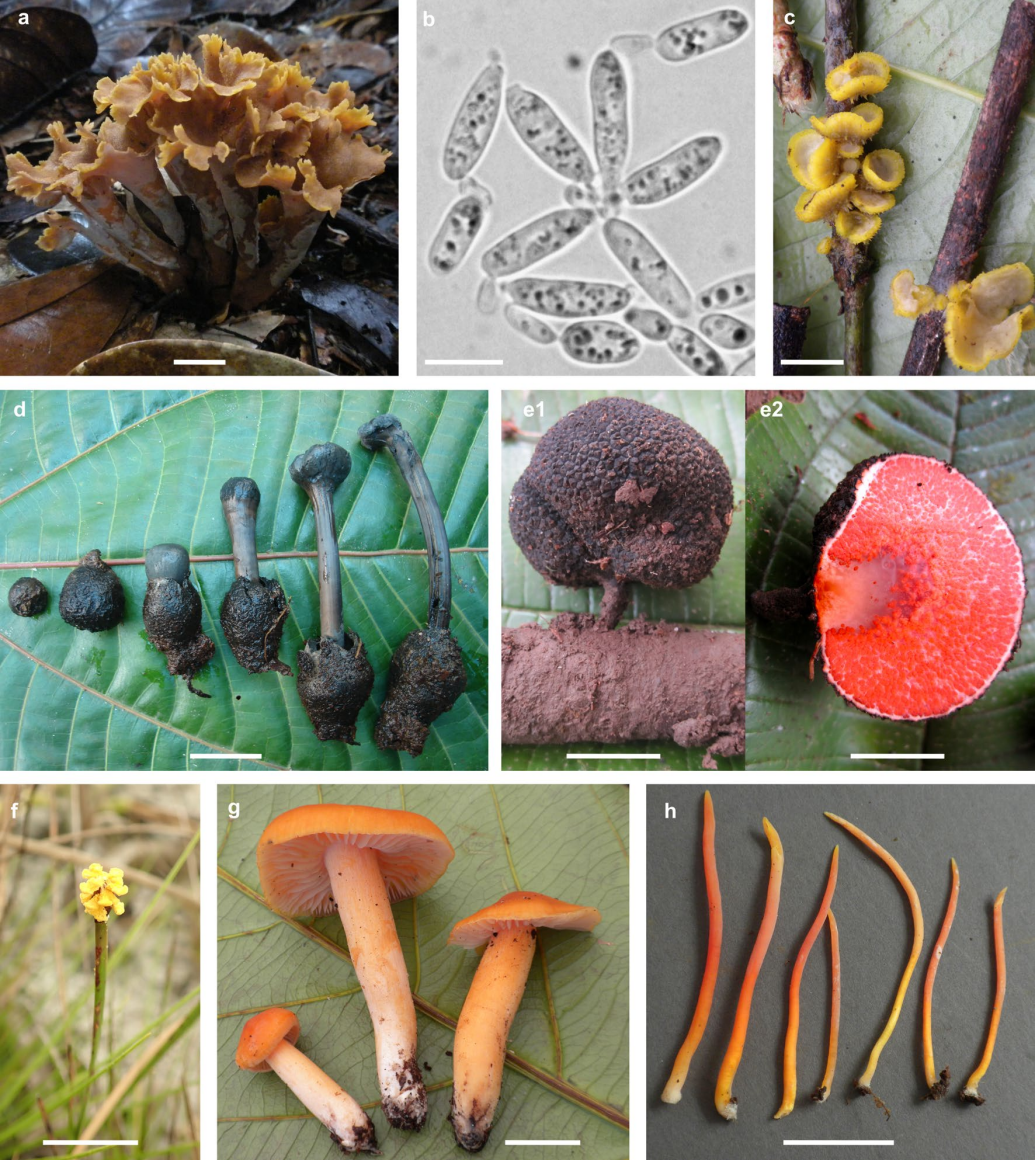
New, unusual, and endemic tropical taxa from the UPRS. *Clavulina craterelloides*, unique morphology for this genus **a**, *Meredithblackwellia eburnea*, new genus **b**, *Craterellus pleurotoides*, unique morphology for this genus **c**, *Pseudotulostoma volvata*, new genus **d**, *Guyanagaster necrorhizus*, new genus **e1–2**, *Fusarium xyrophilum*, new life history strategy for this genus **f**, *Guyanagarika pakaraimensis*, new genus **g**, *Hygrocybe* sp., unique morphology for this genus **h**. Scale bars a, d–h 2 cm; b 10 µm; c 1 cm

In the UPRS, 128 new species and 10 new genera have been described (Table S1). This is approximately one quarter of the estimated new species from this system (Blackwell 2011). New genera include *Guyanagarika*, a robust mushroom-forming ECM genus (Sánchez-García et al. 2016), *Meredithblackwellia*, a monotypic yeast genus (Toome et al. 2013), and *Guyanagaster*, an unusual sequestrate taxon related to *Armillaria* (Henkel et al. 2010; Koch et al. 2017) (Fig. 2). All are putatively endemic to the UPRS. The UPRS is likely not unique in having many novel species and genera. A LTS in the Dja Biosphere Reserve of Cameroon from 2014–2019 has thus far resulted in the description of at least one new genus and 18 new species in eight genera—all only known from their type localities (Table S3). Additionally, a LTS in Thailand has resulted in the description of over 500 new species with > 80% of species collected in conspicuous genera such as *Agaricus* and *Amanita* being new (Hyde et al. 2018).

#### Unique morphologies and expanding higher-rank concepts

The majority of fungal species have been described from non-tropical regions (Fig. 1), and consequently, most higher-rank diagnoses are based on characteristics of temperate taxa. This can be problematic in tropical systems where attempts to place taxa into known higher-level groups can be confounding based on current diagnoses. For example, the species of the genus *Clavulina* were primarily known as fleshy, ECM fungi that produced coralloid sporocarps. Work in UPRS uncovered a wealth of species that range in sporocarp morphology from resupinate, effuso-coralloid, sub-globose, and sub-cerebriform to craterelloid (Henkel et al. 2005, 2011; Thacker and Henkel 2004; Uehling et al. 2012a, 2012b) (Fig. 2). Of the 51 new species of *Clavulina* that have been described globally since 2000, 15, or 29%, are from the 6-ha study site at UPRS (Table S4). Nine of these 15 new species were also later reported from Colombia (Vasco-Palacios and Boekhout 2022).

Additional examples of taxa with unique morphologies include *Amauroderma coltricioides*, the first known species in *Ganodermataceae* with unornamented spore walls, originally identified in the field as a *Coltricia* species (Aime et al. 2003). *Craterellus pleurotoides*, which produces gregarious, astipitate funnel-shaped basidiomata on sticks and litter, was originally identified in the field as a member of *Pezizales*, but is now the first known pleurotoid member of *Cantharallales* (Henkel et al. 2006a). In fact, pleurotoid forms of taxa that are primarily known as stipitate–pileate in temperate regions seem to be a common adaptation in tropical fungi, especially within *Russulales* (Henkel et al. 2000; Miller et al. 2000, 2002; Buyck and Horak 1999).

### Ecological and functional diversity

#### Multi-domain interactions

While the documentation of new taxa is important, LTS can also provide data for assessing ecological and functional diversity. For example, the discovery of the new sequestrate genus and species *Guyanagaster necrorhizus* in UPRS (Henkel et al. 2010) led to questions about how it dispersed its spores because it lacked traits possessed by temperate sequestrate fungi for wind, rain, or mammal dispersal. Multi-year surveys, and a combination of proteomics, genomics, population genetics, and nitrogen fixation assays demonstrated that wood-feeding termites feed on the gleba of mature *G. necrorhizus* sporocarps. During feeding, mature basidiospores adhere to the termite exoskeletons for subsequent dispersal to woody substrates (Koch and Aime 2018). This is the first known instance of selective sporocarp feeding by termites, and one of the only examples of nitrogen fixation within a basidiocarp. Additional studies showed the fungus hosts nitrogen-fixing bacteria within the sporocarp to supplement termite diets, uses fermentation to produce the energy for nitrogen-fixation, and likely maintains an anoxic environment through the production of a thick, impervious exoperidium (Koch et al. 2021). The secretion of mucilage when the spores of *G. necrorhizus* are mature is also hypothesized to ensure spore adhesion to termite exoskeletons (Koch et al. 2021).

#### Fungi and bird nests

LTS in both the UPRS and the Dja Biosphere Reserve, Cameroon have helped to elucidate the interactions between fungal rhizomorphs and avian fauna. Rhizomorphs are autonomous vegetative structures produced by many species in the *Marasmiineae*. In tropical rainforests, rhizomorphs are most common aboveground. They form wiry webs throughout the canopy and play a crucial role in aboveground decomposition (Hedger et al. 1993) while providing food and shelter for arthropods (Snaddon et al. 2012). One estimate suggests that 70% of arthropods are supported by these rhizomorph networks in the lower canopy zones (Snaddon et al. 2012). Recent studies, however, have shown that aerial rhizomorphs are also a component of bird nests in both the neotropics and paleotropics and involve at least 27 rhizomorph-forming fungal species (César et al. 2018; Koch et al. 2018, 2020; Elliott et al. 2019). There is now increasing evidence that birds are selective in incorporating rhizomorphs of different fungal species for different parts of nest construction (Koch et al. 2020), and that selective advantages include structural support (Freymann 2008; Koch et al. 2020), and possibly antibiotic production for parasite control (Aubrecht et al. 2013; Koch et al. 2020). One hypothesis for why the enigmatic fungus, *Brunneocorticium corynecarpon* (*Marasmiaceae*), has never been observed to produce any means of sexual or asexual reproductive structures is that it is adapted for vegetative dispersal by birds.

#### Floral and fungal mimicry

Another unique interaction discovered in the UPRS is floral mimicry, or pseudoflower formation by the recently discovered and described fungus, *Fusarium xyrophilum* (Laraba et al. 2020a). Pseudoflower formation by fungi was thus far only known in temperate rust species (Batra and Batra 1985; Roy 1993; Raguso and Roy 1998; Pfunder and Roy 2000; Naef et al. 2002) by the modification of plant tissue. In contrast, pseudoflowers formed by *F. xyrophilum* are entirely composed of fungal tissue, mimicking visual and olfactory cues of true flowers to attract insect pollinators (Laraba et al. 2020b).

Tropical LTS also offer examples of the opposite phenomenon, when plants mimic a fungus to enhance pollination or dispersal. Dracula orchids, restricted to mountainous habitats of tropical America, have evolved similar visual and olfactory characteristics as mushrooms for deceptive pollination by flies seeking places to lay their eggs (Kaiser 2006; Dentinger and Roy 2010; Endara et al. 2010). In a LTS in Ecuador, Policha et al. (2016, 2019) showed that flies were attracted to fungus-mimicking flowers by both olfactory and visual cues and that the flies suffered at least some fitness reduction in using flowers instead of mushrooms to lay their eggs. Flowers that mimic fungal sporocarps are rare, and so far, all examples are known only from the tropics. This includes the understory tree, *Duguetia cadaverica*, found in humid forests in the Guianas, which produces flowers that mimic stinkhorns in both morphology and scent to deceptively attract stinkhorn-associated insects (Teichert et al. 2012).

### Biogeography

#### Quantifying disparities in DNA sequence data

**Fig. 3:**
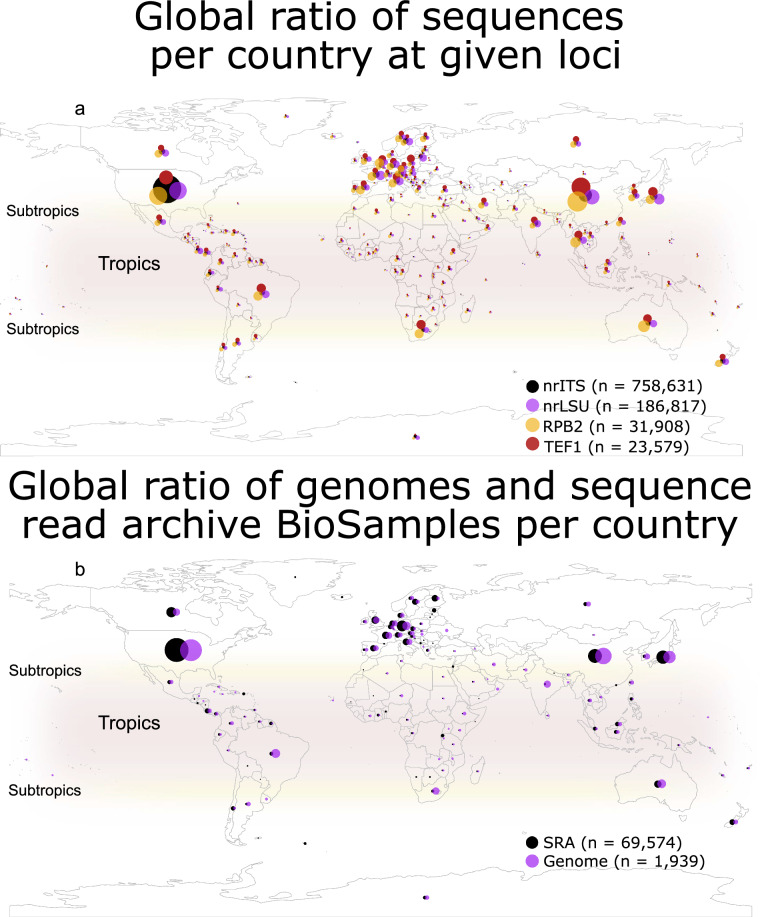
NCBI nucleotide, Sequence Read Archive BioSample, and genome data on tropical and non-tropical fungi. Binned counts of nucleotide sequences at nrITS, LSU, *TEF1*, and *RPB2* loci **a** and Sequence Read Archive (SRA) BioSample and genome sequences per country in NCBI **b** as of November 2020. Sizes of circles represent 10 binned data classes unique to each data set. The largest bubbles represent 48, 18, 21, and 17%, of nrITS, LSU, *TEF1*, and *RPB2* sequences, 28% of genomes, and 34% of SRA BioSamples, respectively. The smallest bubbles represent 1.32e^−4^%, 5.35e^−4^%, 4.22e^−3^%, and 3.13e^−e^% of nrITS, LSU, *TEF1*, and *RPB2* sequences, 5.16E^−2^% of genomes, and 1.44e^−3^% of SRA BioSamples, respectively

With the development of HTS over the last 20 years, microbial biogeography has quickly advanced to test theories developed in plants and animals on primarily microbial organisms, such as fungi (Dickey et al. 2021). DNA sequence data are crucial to compare individual taxa across global scales, improve range assessments, determine endemism, and perform other biogeographical analyses. To quantify the global distribution of public fungal genetic and genomic data, we examined popular loci used for fungal barcoding and phylogenetics–ITS, LSU, *TEF1*, *RPB2*–as well as BioSamples in the Sequence Read Archive and genome sequencing studies in the NCBI archives (methods in Additional file 1). We found biases towards predominantly non-tropical locations such as the United States, Europe, and China in all datasets (Fig. 3a, b). Biogeographical information bias is often referred to as the Wallacean shortfall (Hortal et al. 2015) and has previously been identified in understudied groups of fungi such as *Leotiomycetes* and *Laboulbeniomycetes* (Quandt and Haelewaters 2021; Haelewaters et al. 2024a, b). Less DNA sequence data from both sporocarps and environmental samples in tropical regions limits biogeographical knowledge and therefore also hampers understanding of fungal diversity, evolution, and conservation.

#### Biodiversity, distribution, and endemism of ectomycorrhizal *fungi*

Ectomycorrhizal fungi were traditionally hypothesized to be insignificant in tropical habitats, whereas arbuscular mycorrhizal fungi were archetypal (Redhead 1968; Thomazini 1974; Bereau et al. 1997). However, this paradigm was informed by the disproportionate amount of ECM research conducted in non-tropical habitats as compared to tropical habitats. LTS in tropical ecosystems over the last decades have dispelled this notion and provide evidence of the robust diversity and ecological significance of ECM in tropical habitats (e.g., Alexander 2006; Diédhiou et al. 2010; Peay et al. 2010; Tedersoo et al. 2010a; Smith et al. 2011; Vasco-Palacios 2016; Corrales et al. 2022). Although important, global HTS sequencing of soils has repeatedly found that ECM fungal diversity is lower in the tropics than in temperate and boreal ecosystems (Tedersoo and Nara 2010; Tedersoo et al. 2014).

In the UPRS, approximately 172 species of ECM taxa have been collected from sporocarps, where they are primarily associated with a single host species (Henkel et al. 2012). Eighty-four of these species have been described to date (Table S1) and sequencing of root tips in this region suggests that at least 250 ECM-forming species exist in this system with an additional two host tree species (Smith et al. 2011). Sporocarp collections from the UPRS represent approximately 30% more species diversity of ECM fungi than recovered in a 21-year plot study of similar size in a temperate forest in Switzerland with seven ECM host trees (Straatsma et al. 2001). This comparison, the presence of putative endemic ECM genera in the UPRS, such as *Guyanagarika*, and the possibility that HTS datasets from tropical ECM systems do not account for the heterogeneous distribution of ECM host plants in tropical forests (Morris et al. 2009; Diédhiou et al. 2010; Peay et al. 2010; Tedersoo and Nara 2010; Tedersoo et al. 2010b) signal there may be more diversity present in these systems than is revealed by current HTS datasets.

Conversely, recent studies have shown that many ECM species occurring with *D. corymbosa* in Guyana also occur in Colombia with different hosts. For example, *Pseudotulostoma volvata* (Fig. 2d), a fungus in *Eurotiales* that produces tall, fleshy ascomata with a stipe and volva (Miller et al. 2001) and is an ECM fungus of *D. corymbosa* (Henkel et al. 2006b) was later shown to associate with *Pseudomonotes tropenbosii* in Colombia (Vasco-Palacios 2016) and *Aldina heterophylla* in Brazil (Komura et al. 2021). Additionally, species of ECM fungi from a white sand forest dominated by *D. uaiparuensis* and an *Aldina* sp. in Colombia’s western Amazonia had a 42% overlap with species reported from Guyana (Vasco-Palacios et al. 2018). At least 60% of *Clavulina* spp. reported from Guyana are also found in Colombian Amazonia forests dominated by *P. tropenbosii* (Vasco-Palacios and Boekhout 2022). Although broad distributions of many lowland ECM-forming fungi among different hosts may suggest lower diversity, Colombia still has a putative endemism rate of 18% for ECM fungi, most being found in the Andean region (Vasco-Palacios et al. 2022). Sampling ECM fungi from more host trees across broader geographic ranges will help clarify whether tropical ECM fungi are mostly widespread generalists or have narrower ranges and host preferences.

#### Endemism of non-ECM *fungi* and *fungi* on islands

Endemism of soil fungi has been found to be highest in tropical regions in HTS studies (Tedersoo et al. 2022) and LTS can help discover species that are found nowhere else in the world. In the UPRS, the genus *Guyanagaster* consists of two species, *G. lucianii* and *G. necrorhizus*, that are distributed ~ 125 km apart with no evidence of overlapping ranges (Koch et al. 2017). This is suggestive of specialized adaptation of this genus to the region (Koch and Aime 2018). Another putative endemic species in the UPRS is *Meredithblackwellia eburnea*, a rare, monotypic, distinctive yeast with a rosette budding pattern (Fig. 2). Despite more than a decade of phylloplane isolations in this region, only a single isolate representing the holotype was ever recovered. Querying internal datasets of HTS data from leaf litter and root samples from the UPRS (R.A. Koch Bach and M.C. Aime unpublished), *M. eburnea* was found in 26 out of 3.7 million reads, being one of 4583 OTUs present in this dataset after quality processing. To date, no collection-based or HTS studies from any other region of the world have uncovered *Guyanagaster* spp. or *M. eburnea* outside of the Pakaraima Mountains, supporting the hypothesis that these species represent true endemics with limited range and dispersal.

LTS of macrofungi in the Hawaiian Islands more than tripled the number of known agarics from this archipelago from 1990 to the early 2000s (Hemmes and Desjardin 2002). Eighty-eight percent of the native species were considered endemic to the Hawaiian Islands, a number approximated by later studies (Mueller et al. 2007). Of Hawaiian-endemic *Agaricomycetes*, 18% are *Hygrophoraceae*. Similarly, of the 63 *Hygrophoraceae* taxa in the Greater Antilles, 36% have limited distributions (Cantrell et al. 2001) and the study of *Hygrophoraceae* once thought to be widespread throughout the Antilles and South America show that these taxa are instead complexes of regional endemics (D.J. Lodge, pers. comm.).

Another island system with potentially high endemism of macrofungi is São Tomé and Príncipe. Desjardin and Perry (2022) report 60 putative endemic *Agaricomycetes* from these islands, although the authors cautioned that this is a preliminary estimate due to lack of knowledge of fungal biodiversity on nearby continental Africa. Indeed, many supposedly-endemic species lack DNA sequence data to compare with public databases. For example, only 18% of putative endemic Hawaiian Agaricomycetes species have reference DNA sequences (Stallman et al. 2023). Although more studies incorporating DNA sequence data may lower endemism estimates, such as the case with putative endemic ECM fungi in Guyana later found in Colombia (Vasco-Palacios and Boekhout 2022), the opposite is often true. For example, multiple studies of lichens have shown that endemism estimates increase when DNA sequence data is incorporated in both continental (Vasco-Palacios et al. 2022) and insular (Moncada et al. 2020) systems due to cryptic or semi-cryptic species that were not previously recognized.

#### Documentation of emerging diseases

Emerging fungal pathogens threaten the stability of both natural and anthropogenic ecosystems and are therefore a crucial topic of study. They can cause new diseases, shift hosts, have an unusually high incidence, and exhibit fast geographic expansion (Anderson et al. 2004; Fisher et al. 2012; Corredor-Moreno and Saunders 2020). Although origins of most emerging fungal pathogens are unknown, many are suspected to derive from the tropics (Nnadi and Carter 2021). Examples of fungal diseases that have emerged in tropical areas and become threats in temperate areas are chytridiomycosis (*Batrachochytrium dendrobatidis*) in amphibians (Scheele et al. 2019; Fisher and Garner 2020) and tar spot (*Phyllachora maydis*) in corn (Ruhl et al. 2016).

One of the advantages of LTS is the ability to potentially detect and document new pathogens before they become more broadly dispersed. For example, *Xylaria karyophthora*, a pathogen of the seeds of *Chlorocardium* spp. (*Lauraceae*), was first discovered during LTS in Guyana. This was the first record of a putative fungal pathogen associated with a commercial timber species in the Guyanese rainforest system. Approximately 80% of dispersed seeds in both natural and logged forests are affected, limiting germination and seedling recruitment (Husbands et al. 2018; Husbands and Aime 2018). Early identification and reporting of new and emerging disease epidemics can improve disease management outcomes (Parnell et al. 2015) and this could be particularly important for *Chlorocardium* spp., which are major components of local, regional, and international forestry markets. Cultures of *X. karyophthora* have already led to the discovery of a novel secondary metabolite, karyochalasin (Lambert et al. 2023), and will facilitate future studies on this fungus.

### Hypothesis testing

#### Sequestration

Sequestration describes a morphological transition in which a species with an exposed hymenium and spores, adapted for forcible discharge, speciates into one with an enclosed hymenium, and spores that are passively discharged. This process has occurred independently in many lineages within *Ascomycota* and *Basidiomycota* with epigeous sporocarps (e.g., Hibbett et al. 1997; Moreno et al. 2014). The stimuli driving the convergent evolution of sequestrate forms are not clearly understood but have traditionally been hypothesized to be an evolutionary adaptation to protect spores from desiccation in cold and arid regions (e.g., Thiers 1984; Miller and Miller 1988;). This hypothesis is supported by the high diversity of sequestrate fungi from arid areas (e.g., Lebel and Syme 2012; Sheedy et al. 2016), seasonally dry North American regions (e.g., Fogel and States 2001; Trappe et al. 2009), and temperate Australia (e.g., Bougher and Lebel 2001; Sheedy et al. 2016).

The paucity of published records of sequestrate taxa from the tropics has also contributed to the belief that sequestration was driven by temperate and boreal climates. However, this belief is eroding as both LTS from Guyana and Cameroon (Henkel et al. 2010; Castellano et al. 2012, 2016a, 2016b; Smith et al. 2015) and other studies in tropical America, Africa, and Asia (Orihara and Smith 2017; Elliott et al. 2020; de la Fuente et al. 2023) are uncovering a large consortium of novel sequestrate lineages and species. Therefore, it is likely that temperature and moisture level are not the only drivers of this syndrome. An alternative hypothesis suggests neoteny or progenesis may lead to sequestration instead of requiring a gradual, selection driven process (Kuhar et al. 2023).

#### Island biogeography hypotheses

Documenting fungi in discrete locations for long periods can lead to species checklists that are useful for fungal conservation initiatives, monitoring, and comparative studies of fungal diversity, including biogeography (Piepenbring et al. 2020). Part of the theory of island biogeography suggests that larger, more isolated islands are part of a ‘radiation zone’ and should have higher numbers of endemic species (MacArthur and Wilson 1967; Whittaker et al. 2008). Using checklists of *Agaricomycetes* from seven oceanic islands and archipelagos, Stallman et al. (2023) found a positive correlation between endemism percentage and island size and distance to mainland.

Beyond checklists, thorough sporocarp collecting from discrete locations may lead to many collections of the same species that can be used in biogeography and population genetics studies. In the Hawaiian Islands, Keirle et al. (2011) tested the progression rule (Funk and Wagner 1995) whereby species are hypothesized to colonize the geologically oldest island in a volcanic archipelago, then disperse to progressively newer, emerging islands as they appear. Using 120 collections of the putative endemic species *Rhodocollybia laulaha* spanning 20 years across 28 collecting sites and three different islands within the archipelago, they did not find evidence *R. laulaha* followed this dispersal pattern.

#### Foraging ascomycete hypothesis and viaphytism

The foraging ascomycete hypothesis (Carroll 1999) posits that some *Ascomycota* species have life history strategies in which they spend substantial periods of time as endophytes to avoid disadvantageous climatic conditions. They also use their leaf hosts as an additional method of dispersal. When leaves senesce and fall to the ground, endophytes can colonize woody substrates where they may produce saprotrophic reproductive structures. This hypothesis has been tested in LTS in the Ecuadorian cloud forest of Los Cedros (Vandegrift et al. 2023).

Thomas et al. (2016) showed that there is some support for release from environmental constraints in the endophytic life stage of *Xylaria* spp. regarding water availability, and found spatial coupling between two *Xylaria* spp. as both endophytes and sporocarps. Nelson et al. (2020) showed that it was possible for a variety of endophytes of both *Ascomycota* and *Basidiomycota* to colonize wood via leaves, confirming this phenomenon with far greater taxonomic breadth than *Xylaria* (as shown in Thomas et al. 2016), and introducing the term viaphyte to refer to fungi with this life history strategy. Finally, Thomas et al. (2020) provide a model of the tradeoffs made by fungi engaging in this life history strategy, providing theoretical grounding that it can be advantageous for some fungi, particularly under conditions that allow long-lasting endophytes in the canopy. This provides an opportunity for additional hypothesis testing as the model suggests viaphytism should be more common in tropical areas with long-persisting leaves versus non-tropical deciduous forests with annual turnover.

### Conservation

#### Threats to fungi and comparisons between tropical, non-tropical, and non-fungal taxa

Loss of biodiversity is one of the most critical environmental problems, threatening valuable ecosystem services and human well-being (Daily and Matson 2008; Mace et al. 2012; Ehrlich and Ehrlich 2013). This problem is most acute in tropical rainforests, which harbor more than half of all known species, but are being depleted faster than any other ecosystem (Myers 1988). Changes in land use leading to habitat loss and degradation are predicted to have the largest negative impact on biodiversity in tropical ecosystems (Sala et al. 2000) and land development was also identified as the top threat to threatened and near-threatened fungi evaluated on the IUCN Red List (Mueller et al. 2022).

**Fig. 4:**
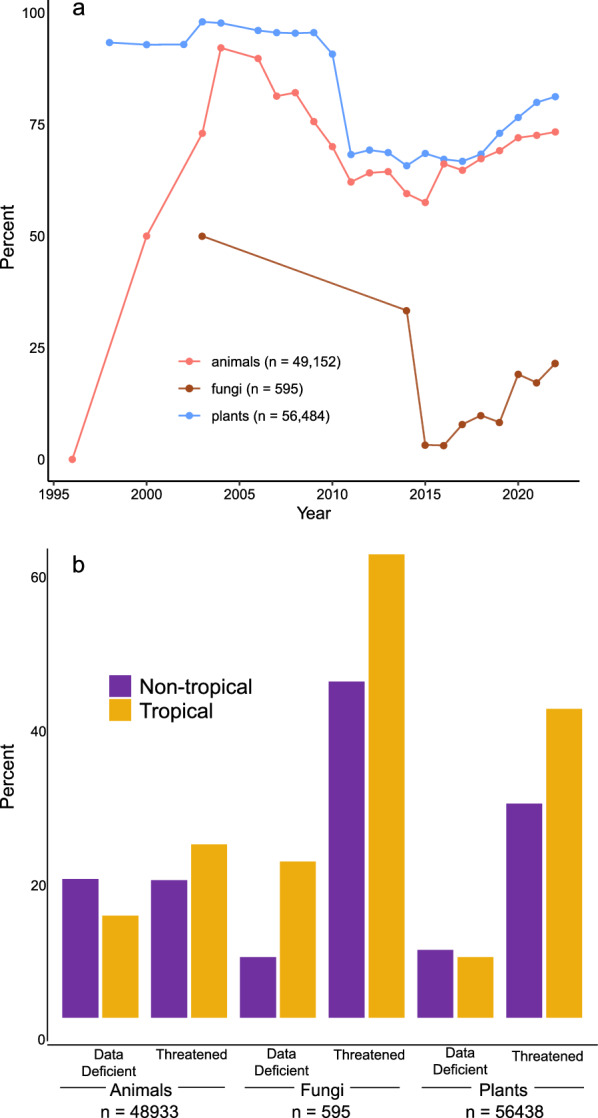
IUCN Red List data on tropical fungi, plants, and animals. Cumulative percent of IUCN evaluations for species of fungi, plants, and animals from tropical habitats **a** and percent fungi, plants, and animals considered threatened or data deficient from tropical and non-tropical locations **b** through June 2023

To examine differences between tropical and non-tropical fungi, plants, and animals, we downloaded IUCN Red List data through June 2023 for all terrestrial organisms in these groups (see methods in Additional file 1). We found the absolute number of fungi evaluated for conservation status by the IUCN is drastically lower than both plants and animals, as shown in other studies (Haelewaters et al. 2024b). We also found that there is a disproportionate number of evaluations for non-tropical fungi (Fig. 4a), a discrepancy noted by Corrales et al. (2022) for ECM fungi, and broadly shown by Niskanen et al. (2023) in their country-by-country analysis. The difference between tropical and non-tropical evaluations skews the opposite direction for plants and animals (Fig. 4a). We also found that data-deficient evaluations were more than twice as common in tropical fungi than non-tropical fungi, whereas in plants and animals it is more common for non-tropical species to have data-deficient evaluations (Fig. 4b). Finally, tropical fungi are the most frequently evaluated as threatened (critically endangered, endangered, or vulnerable) among the organismal groups examined (Fig. 4b).

While there is a discrepancy between global conservation data available on tropical and non-tropical fungi, efforts by the Global Fungal Red List Initiative to increase evaluations in targeted regions have been successful. For example, workshops in (tropical and non-tropical) South America have greatly increased the number of evaluations from this region (Mueller et al. 2022). Therefore, hosting workshops in tropical locations may lead to more conservation assessments from these localities.

#### Rare, invasive, and cosmopolitan species

Data from LTS allow researchers both observational and quantitative insights on the species that are present, absent, rare, and common in their system. Without repeated sampling, data on rarity or population shifts may be lacking or not available when conditions change. For example, LTS of agarics from the Hawaiian Islands led to detailed information on species ranges and prevalence, including many endemic *Hygrophoraceae* species that are rare or have restricted ranges (Desjardin and Hemmes 1997; Hemmes and Desjardin 2002). When a non-native pathogen arrived that began killing the dominant tree species within these limited ranges (Barnes et al. 2018), decades of observation meant mycologists could quickly evaluate if the co-occurring *Hygrophoraceae* species would also be threatened. Indeed, these data helped with five IUCN evaluations, including multiple ‘endangered’ assessments (e.g., Vellinga 2017, 2019).

In addition to recognizing the rarity of species native to study sites, LTS can also help determine when newer, non-native species have established, and provide insights on putatively cosmopolitan species. For example, Hemmes et al. (2018) documented newly established species since the publication of a field guide to the Hawaiian Islands (Hemmes and Desjardin 2002), including a species in the invasive *Favolaschia calocera* complex (Vizzini et al. 2009; Zhang et al. 2023). The cosmopolitan fungus *Schizophyllum commune* is common and abundant in the Caribbean and South America (James et al. 2001; James and Vilgalys 2008) yet has never been collected within the study plots of UPRS. Additionally, no sequences of *S. commune* can be identified in HTS litter samples of UPRS (R.A. Koch Bach and M.C. Aime unpublished), suggesting there are regional limits to establishment even for globally dispersed, putatively cosmopolitan fungi.

#### Using non-traditional data sources to study global change

Global anthropogenic changes are already occurring, and therefore “baseline” data of reference systems may no longer exist. However, it is still critical to acquire as many data as possible, particularly from habitats that are relatively preserved from human destruction. While systematic LTS in relatively undisturbed habitats are one option, innovative strategies can be used now to access baseline biodiversity data and track changes over time.

Non-fungal organisms and abiotic objects can provide resources to study fungal diversity across time and space. For example, Tipton et al. (2019) used saved air filters from the Mauna Loa observatory in Hawai’i to examine the diversity of the aerial mycobiota annually for 13 years. Plants in herbaria and their associated fungi can be used to track pathogens (Lang et al. 2019), fungal hyperparasites (Gómez-Zapata et al. 2024), endophytes (Datlof et al. 2017), and potentially other fungal symbionts through time and space. Likewise, preserved insects (dried and pinned or in DNA) have been used to infer historical range and incidence of fungal parasites on a given host over time (Haelewaters et al. 2017). Therefore, filters, natural history collections, and any other objects encountering fungal particles could be used to study fungal diversity over time.

Attempting to replicate studies or species inventories that were completed pre-disturbance at the same location is another possibility. For example, Kaishian (2021) sampled Lake Eustis in Florida for insects carrying *Laboulbeniales* 121 years after Roland Thaxter’s inventory in the area (Thaxter 1908, 1924, 1931). The recent survey compared Thaxter’s original inventory with species present at Lake Eustis, an urbanized area, and the Emeralda Marsh Conservation Area (EMCA), which was restored and protected. The study found that 13 of 27 species originally recorded were found within the EMCA, while only one species was found at Lake Eustis. These results suggest that the EMCA was at least partially effective at protecting fungal biodiversity.

In addition to making new collections, historical data of fungi accessioned in herbaria from MyCoPortal (https://www.mycoportal.org/) and observations of fungi from iNaturalist (https://www.inaturalist.org/) can be used to examine fungal diversity over time. Although citizen science data may have biases (Geldmann et al. 2016) and species may not be identified correctly (McMullin and Allen 2022), these data can be useful to observe broad trends, such as phenology of sporocarp production. For example, some tropical localities, such as cloud forest environments, have above-freezing temperatures and high-precipitation year round, leading to uncertainty about whether sporocarp production is even throughout the year, or peaks in diversity or abundance occur annually during particular periods as in non-tropical systems. Using a checklist of *Agaricomycetes* species (Mueller et al. 2007) with observation and collection data from iNaturalist and MyCoPortal, Stallman and Robinson (2022) found sporocarp production of *Agaricomycetes* spp. in the Hawaiian Islands varied throughout the year. Richness and abundance were positively correlated with increased monthly rainfall on only two of the four islands examined and were not even throughout the year. This indicates that even in ‘aseasonal’ tropical areas, richness and abundance of sporocarp production may vary throughout the year and should be a consideration when planning surveys.

**Fig. 5:**
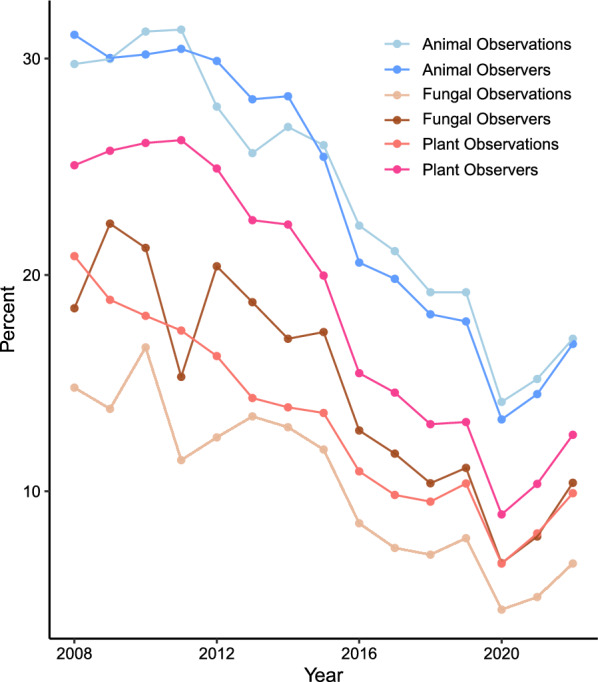
iNaturalist.org data on tropical fungi, plants, and animals. Percent observations or observers occurring from tropical areas for fungi, plants, and animals through December 31, 2022. For fungi, n = 7,817,544 observations and 906,838 observers; for plants, n = 50,849,281 observations and 2,535,549 observers; for animals, n = 64,390,584 observations and 2,772,329 observers

Despite the potential for citizen science data to augment tropical fungal datasets and be used for conservation or other purposes, we found the use of iNaturalist has much lower representation from tropical regions (Fig. 5, methods in Additional file 1). Fungi, but also plants and animals, have lower percentages of observations and observers from tropical regions with general trends showing this discrepancy worsening until the year 2020. Although trends are shared between fungi, plants, and animals, tropical fungi still have the lowest percentages among observations (annual average 10% vs 13% in plants and 24% in animals in 2008–2022) and observers (annual average 15% vs 19% in plants and 24% in animals in 2008–2022).

## Conclusions

This paper argues for the necessity of tropical LTS to better understand fungi. We acknowledge that much of the knowledge of tropical fungal biodiversity is granted to researchers from non-tropical regions with access to more financial resources than mycologists based in tropical areas. LTS cannot address all aspects of this complex issue (Dahdouh-Guebas et al. 2003; Ryan et al. 2019; Minasby et al. 2020) but working repeatedly in the same location increases the potential for making personal connections, training students, and collaborating with local and indigenous researchers. While this cannot ameliorate all the problems associated with an imbalance of resources or “helicopter” science (Haelewaters et al. 2021a), building equal collaborations with individuals with local and/or indigenous knowledge often improves the science itself (Ward-Fear et al. 2019; Copete et al. 2023) and can increase capacity in localities with limited resources (Gryzenhout et al. 2012; Piepenbring and Yorou 2017). Additionally, we suggest working with local collaborators to address important logistical issues for field-based studies such as cost, safety, and methodology that are not addressed in this review and will vary by locality and study goals.

LTS in the tropics have improved our understanding of alpha, ecological, functional, and geographic diversity of fungi. While different LTS may have varying goals and generate fungal biodiversity data from field-work or alternative sources, this variety often is complementary and improves the scope of our knowledge of fungi. We hope mycologists continue building on this foundation to reduce the disparities highlighted here in species descriptions, DNA sequence data, conservation data, and citizen science data. Only by incorporating ample data from tropical environments will we be able to understand Kingdom Fungi at a global scale.

## Supplementary Information

Additional file 1. Provides methods for analyses completed.

Additional file 2. Provides MycoBank data used to create Fig. 1.

Additional file 3. Provides ITS nucleotide data from NCBI used to create Fig. 3.

Additional file 4. Provides LSU nucleotide data from NCBI used to create Fig. 3.

Additional file 5. Provides EF1 nucleotide data from NCBI used to create Fig. 3.

Additional file 6. Provides RPB2 nucleotide data from NCBI used to create Fig. 3.

Additional file 7. Provides genome data from NCBI used to create Fig. 3.

Additional file 8. Provides BioSample data from NCBI used to create Fig. 3.

Additional file 9. Provides IUCN Red List data on animals used to create Fig. 4.

Additional file 10. Provides IUCN Red List data on fungi used to create Fig. 4.

Additional file 11. Provides IUCN Red List data on plants used to create Fig. 4.

Additional file 12. Provides iNaturalist.org data used to create Fig. 5.

Additional file 13. Table S1. New species and genera originating from long-term studies in Guyana.

Additional file 14. Table S2. Papers published with data from long-term studies in Guyana.

Additional file 15. Table S3. New species and genera originating from long-term studies in Cameroon.

Additional file 16. Table S4. *Clavulina* species described since 2000

## Data Availability

All data generated or analyzed during this study are included in this published article and its supplementary files.

## Abbreviations

ECM: Ectomycorrhizal
EMCA: Emeralda marsh conservation area
HTS: High-throughput sequencing
ITS: Internal transcribed spacer barcode region consisting of spacers ITS1 and ITS2 and the conserved 5.8S gene
IUCN: International union for conservation of nature
LSU: Large subunit of the nuclear ribosomal RNA gene
LTS: Long-term studies
NCBI: National Center for Biotechnology Information
OTU: Operational taxonomic unit
RPB2: Second largest subunit of the RNA polymerase II gene
TEF1: Translation elongation factor 1α
UPRS: Upper Potaro River Study
