# Supplementary material for: The contribution of tropical long-term studies to mycology
Source: IMA Fungus. 2024 Nov 11;15:35. doi: 10.1186/s43008-024-00166-5 (PMC11552369; doi:10.1186/s43008-024-00166-5)
Supplement: Supplementary file 1 — Additional file 1. Provides methods for analyses completed. [file 43008_2024_166_MOESM1_ESM.docx]

**Methods for MycoBank analysis (Fig. 1)**

Fungal names deposited in MycoBank were received in November 2020 and consisted of 444,929 entries. We removed all illegitimate and duplicate names, new combinations, and entries lacking year or locality metadata, then assigned taxa to one of three categories: tropical, non-tropical, or unknown. Tropical was assigned if the locality of origin was contained between the Tropic of Capricorn and the Tropic of Cancer, non-tropical was assigned if the locality of origin fell outside these limits or was highly probable, and unknown was assigned in all other cases. For example, a locality in the country Cuba was assigned as tropical, Sweden was assigned as temperate, and a locality in the United States or France was inferred as temperate unless contradicted by more precise location information (e.g., origin in Hawai'i or Réunion). This resulted in 43,461 tropical, 89,456 temperate, and 233,024 unassignable names.

**Methods for NCBI analysis (Fig. 3).**

Nucleotide data from fungal ITS, LSU, RPB2 and TEF1 regions, fungal genome assemblies, and BioSamples from sequence read archive (SRA) projects involving fungal nrITS reads were downloaded from NCBI (NCBI Resource Coordinators 2018) via the srchhk and edirect tools (Schoch 2020) in November 2020. Datasets were manually inspected to remove entries lacking appropriate metadata to assign a country of origin and identify the study organism to kingdom Fungi.

For nucleotides, we checked the nrITS and nrLSU loci for model organisms in the Ascomycota (*Saccharomyces cerevisiae*) and Basidiomycota (*Coprinopsis cinerea*) to help determine a cutoff for removing model organisms more likely to result from cultures and laboratory provenance that may not have originated in the country reported. We then took the smallest number of sequences reported from a model organism at that locus (*C. cinerea* in both cases), doubled it to account for population genetics studies or species that may be widespread and/or sequenced more frequently, then removed all sequences from taxa with more than this number of entries. This resulted in removing all sequences with more than 100 representatives at the ITS locus and all sequences with more than 48 representatives at the LSU locus. Although the RPB2 and TEF1 loci did not have sequences for these model organisms, we decided to exclude all taxa with more than 48 sequences, setting the number determined at the LSU locus as a baseline. Duplicate taxa names that were identified at a level above species (e.g., *Suillus* sp. or uncultured *Suillus*) were retained.

For genomes, we only considered a single genomic assembly for each species, selecting the first entry appearing in our search, and excluding all others. For SRA BioSamples, all records were used.

After filtering, we assigned 758,631 accessions at the nrITS locus, 186,817 at LSU, 31,908 at RPB2, 23,579 at TEF1, 1,939 genomes, and 69,574 SRA BioSamples to countries and visualized them using the rworldmap v. 1.3-4 package (South 2011) in R (R Core Team 2020).

**Methods for IUCN Red List analysis (Fig. 4).**

To determine whether species evaluated by the IUCN Red List were more likely to occur in tropical or non-tropical habitats, we analyzed data for terrestrial fungi, plants, and animals from the IUCN (IUCN 2020) as of June 6, 2023. IUCN habitat determinations were used to differentiate tropical versus non-tropical ranges (ie. "Forest - Boreal" for non-tropical versus "Grassland - Subtropical/Tropical Dry" for tropical), or taxa occurring in both regions, and eliminate non-terrestrial habitats. In cases where all taxon habitat determinations were ambiguous (ie., "Artificial/Terrestrial - Urban Areas"), the realm was used to assign a tropical versus non-tropical status. See Table 1 below for designations based on IUCN habitat determination and Table 2 for designations based on IUCN realm below. If both the habitat and realm determination were inconclusive, the species was left coded as ‘unknown’ in the analysis. In general, because subtropical and tropical habitats are not differentiated in these determinations, species with subtropical distributions were included in the tropical (or both) category. For calculating percentages in figures, only species assigned to a category were considered in calculating the final percentages (“both” and “unknown” categories were excluded). Conservation status was grouped into data deficient, not threatened (Least Concern, Near Threatened), threatened (Vulnerable, Endangered, Critically Endangered, Extinct, Extinct in the Wild), and unknown (not applicable, unknown, regionally extinct) categories following IUCN assessments (IUCN 2020).

Using our methods, we assigned tropical vs. non-tropical distributions for 595 IUCN-evaluated fungi (excluding 28 both, 2 unknown), 56484 plants (excluding 2381 both, 387 unknown) and 49152 (excluding 3743 both, 321 unknown) animals. The vast majority of these (595 fungi, 56438 plants, and 48933 animals) were also assignable to a conservation status group. Data visualizations were performed using R (R Core Team 2020).

Table 1. Classification of IUCN habitat codes and names to tropical, non-tropical or unknown categories for analysis.

| IUCN Code | IUCN Habitat Name | Category |
| --- | --- | --- |
| 1.1 | Forest - Boreal | non-tropical |
| 1.2 | Forest - Subarctic | non-tropical |
| 1.3 | Forest - Subantarctic | non-tropical |
| 1.4 | Forest - Temperate | non-tropical |
| 1.5 | Forest - Subtropical/Tropical Dry | tropical |
| 1.6 | Forest - Subtropical/Tropical Moist Lowland | tropical |
| 1.7 | Forest - Subtropical/Tropical Mangrove Vegetation Above High Tide Level | tropical |
| 1.8 | Forest - Subtropical/Tropical Swamp | tropical |
| 1.9 | Forest - Subtropical/Tropical Moist Montane | tropical |
| 2.1 | Savanna - Dry | unknown |
| 2.2 | Savanna - Moist | unknown |
| 3.1 | Shrubland - Subarctic | non-tropical |
| 3.2 | Shrubland - Subantarctic | non-tropical |
| 3.3 | Shrubland - Boreal | non-tropical |
| 3.4 | Shrubland –Temperate | non-tropical |
| 3.5 | Shrubland – Subtropical/tropical dry | tropical |
| 3.6 | Shrubland – Subtropical/tropical moist | tropical |
| 3.7 | Shrubland – Subtropical/tropical high altitude | tropical |
| 3.8 | Shrubland – Mediterranean-type shrubby vegetation | non-tropical |
| 4.1 | Grassland – Tundra | non-tropical |
| 4.2 | Grassland – Subarctic | non-tropical |
| 4.3 | Grassland – Subantarctic | non-tropical |
| 4.4 | Grassland – Temperate | non-tropical |
| 4.5 | Grassland – Subtropical/tropical dry | tropical |
| 4.6 | Grassland – Subtropical/tropical seasonally wet/flooded | tropical |
| 4.7 | Grassland – Subtropical/tropical high altitude | tropical |
| 5.1 | Wetlands (inland) – Permanent rivers/streams/creeks (includes waterfalls) | eliminated |
| 5.2 | Wetlands (inland) – Seasonal/intermittent/irregular rivers/streams/creeks | eliminated |
| 5.3 | Wetlands (inland) – Shrub dominated wetlands | eliminated |
| 5.4 | Wetlands (inland) – Bogs, marshes, swamps, fens, peatlands | eliminated |
| 5.5 | Wetlands (inland) – Permanent freshwater lakes (over 8 ha) | eliminated |
| 5.6 | Wetlands (inland) – Seasonal/intermittent freshwater lakes (over 8 ha) | eliminated |
| 5.7 | Wetlands (inland) – Permanent freshwater marshes/pools (under 8 ha) | eliminated |
| 5.8 | Wetlands (inland) – Seasonal/intermittent freshwater marshes/pools (under 8 ha) | eliminated |
| 5.9 | Wetlands (inland) – Freshwater springs and oases | eliminated |
| 5.1 | Wetlands (inland) – Tundra wetlands (inc. pools and temporary waters from snowmelt) | eliminated |
| 5.11 | Wetlands (inland) – Alpine wetlands (inc. temporary waters from snowmelt) | eliminated |
| 5.12 | Wetlands (inland) – Geothermal wetlands | eliminated |
| 5.13 | Wetlands (inland) – Permanent inland deltas | eliminated |
| 5.14 | Wetlands (inland) – Permanent saline, brackish or alkaline lakes | eliminated |
| 5.15 | Wetlands (inland) – Seasonal/intermittent saline, brackish or alkaline lakes and flats | eliminated |
| 5.16 | Wetlands (inland) – Permanent saline, brackish or alkaline marshes/pools | eliminated |
| 5.17 | Wetlands (inland) – Seasonal/intermittent saline, brackish or alkaline marshes/pools | eliminated |
| 5.18 | Wetlands (inland) – Karst and other subterranean hydrological systems (inland) | eliminated |
| 6 | Rocky Areas (e.g., inland cliffs, mountain peaks) | unknown |
| 7.1 | Caves and Subterranean Habitats (non-aquatic) – Caves | unknown |
| 7.2 | Caves and Subterranean Habitats (non-aquatic) – Other subterranean habitats | unknown |
| 8.1 | Desert – Hot | tropical |
| 8.2 | Desert – Temperate | non-tropical |
| 8.3 | Desert – Cold | non-tropical |
| 9.1 | Marine Neritic – Pelagic | eliminated |
| 9.2 | Marine Neritic – Subtidal rock and rocky reefs | eliminated |
| 9.3 | Marine Neritic – Subtidal loose rock/pebble/gravel | eliminated |
| 9.4 | Marine Neritic – Subtidal sandy | eliminated |
| 9.5 | Marine Neritic – Subtidal sandy-mud | eliminated |
| 9.6 | Marine Neritic – Subtidal muddy | eliminated |
| 9.7 | Marine Neritic – Macroalgal/kelp | eliminated |
| 9.8 | Marine Neritic – Coral Reef | eliminated |
| 9.8.1 | Outer reef channel | eliminated |
| 9.8.2 | Back slope | eliminated |
| 9.8.3 | Foreslope (outer reef slope) | eliminated |
| 9.8.4 | Lagoon | eliminated |
| 9.8.5 | Inter-reef soft substrate | eliminated |
| 9.8.6 | Inter-reef rubble substrate | eliminated |
| 9.9 | Seagrass (Submerged) | eliminated |
| 9.1 | Estuaries | eliminated |
| 10.1 | Epipelagic (0–200 m) | eliminated |
| 10.2 | Mesopelagic (200–1,000 m) | eliminated |
| 10.3 | Bathypelagic (1,000–4,000 m) | eliminated |
| 10.4 | Abyssopelagic (4,000–6,000 m) | eliminated |
| 11.1 | Continental Slope/Bathyl Zone (200–4,000 m) | eliminated |
| 11.1.1 | Hard Substrate | eliminated |
| 11.1.2 | Soft Substrate | eliminated |
| 11.2 | Abyssal Plain (4,000–6,000 m) | eliminated |
| 11.3 | Abyssal Mountain/Hills (4,000–6,000 m) | eliminated |
| 11.4 | Hadal/Deep Sea Trench (>6,000 m) | eliminated |
| 11.5 | Seamount | eliminated |
| 11.6 | Deep Sea Vents (Rifts/Seeps) | eliminated |
| 12.1 | Rocky Shoreline | eliminated |
| 12.2 | Sandy Shoreline and/or Beaches, Sand Bars, Spits, etc. | eliminated |
| 12.3 | Shingle and/or Pebble Shoreline and/or Beaches | eliminated |
| 12.4 | Mud Shoreline and Intertidal Mud Flats | eliminated |
| 12.5 | Salt Marshes (Emergent Grasses) | eliminated |
| 12.6 | Tidepools | eliminated |
| 12.7 | Mangrove Submerged Roots | eliminated |
| 13.1 | Sea Cliffs and Rocky Offshore Islands | unknown |
| 13.2 | Coastal Caves/Karst | unknown |
| 13.3 | Coastal Sand Dunes | unknown |
| 13.4 | Coastal Brackish/Saline Lagoons/Marine Lakes | unknown |
| 13.5 | Coastal Freshwater Lakes | unknown |
| 14.1 | Arable Land | unknown |
| 14.2 | Pastureland | unknown |
| 14.3 | Plantations | unknown |
| 14.4 | Rural Gardens | unknown |
| 14.5 | Urban Areas | unknown |
| 14.6 | Subtropical/Tropical Heavily Degraded Former Forest | tropical |
| 15.1 | Water Storage Areas [over 8 ha] | unknown |
| 15.2 | Ponds [below 8 ha] | unknown |
| 15.3 | Aquaculture Ponds | eliminated |
| 15.4 | Salt Exploitation Sites | eliminated |
| 15.5 | Excavations (open) | eliminated |
| 15.6 | Wastewater Treatment Areas | eliminated |
| 15.7 | Irrigated Land [includes irrigation channels] | eliminated |
| 15.8 | Seasonally Flooded Agricultural Land | eliminated |
| 15.9 | Canals and Drainage Channels, Ditches | eliminated |
| 15.1 | Karst and Other Subterranean Hydrological Systems [human-made] | eliminated |
| 15.11 | Marine Anthropogenic Structures | eliminated |
| 15.12 | Mariculture Cages | eliminated |
| 15.13 | Mari/Brackish-culture Ponds | eliminated |
| 16 | Introduced Vegetation | unknown |
| 17 | Other | unknown |
| 18 | Unknown | unknown |

Table 2. Classification of IUCN realm to tropical, non-tropical or unknown categories for analysis (only used for species with an unknown tropical or extra-tropical distribution following assignment based on habitat).

| IUCN Realm | Category |
| --- | --- |
| Afrotropical | tropical |
| Australasian | unknown |
| Indomalayan | tropical |
| Nearctic | non-tropical |
| Neotropical | tropical |
| Oceanian | unknown |
| Palearctic | non-tropical |

**Methods for iNaturalist.org analysis (Fig. 5)**

Data from iNaturalist.org were downloaded November 14, 2023 by filtering observations by the taxon "Fungi" (for Fungi), “Plant,” (for plants) or “Birds,” “Amphibians,” “Reptiles,” “Mammals,” “Fish,” “Molluscs,” “Arachnids,” and “Insects” (for animals) during each calendar year (January 1st - December 31st) from 2008 to 2022 and recording the number of 'verifiable' observations and observers during this time period to obtain total counts. Then, a bounding box was drawn via the map interface to capture all observations from areas between the Tropic of Cancer and the Tropic of Capricorn, and again observations and observers annually were recorded. Percentages of observers and observations were obtained by dividing counts of taxa in the tropics from total annual counts.
