# Supplementary material for: The contribution of tropical long-term studies to mycology
Source: IMA Fungus. 2024 Nov 11;15:35. doi: 10.1186/s43008-024-00166-5 (PMC11552369; doi:10.1186/s43008-024-00166-5)
Supplement: Supplementary file 13 — Additional file 13. Table S1. New species and genera originating from long-term studies in Guyana. [file 43008_2024_166_MOESM13_ESM.docx]

Table S1. New species and genera originating from long-term studies in Guyana. Asterisks denote newly described genera. Complete citations can be found in Table S2.

| **Name** | **Reference** |
| --- | --- |
| *Alboleptonia angustospora* (now *Entoloma niveominutum*) | Henkel et al. (2010a) |
| *Alboleptonia cystidiosa* (now *Entoloma aimeae*) | Henkel et al. (2010a) |
| *Alboleptonia minima* (now *Entoloma henkelii*) | Henkel et al. (2010a) |
| *Amanita aurantiobrunnea* | Simmons et al. (2001) |
| *Amanita calochroa* | Simmons et al. (2001) |
| *Amanita cyanochlorinosma* | Mighell et al. (2019) |
| *Amanita cyanopus* | Simmons et al. (2001) |
| *Amanita fulvoalba* | Mighell et al. (2019) |
| *Amanita guyanensis* | Mighell et al. (2019) |
| *Amanita perphaea* | Simmons et al. (2001) |
| *Amauroderma coltricioides* | Aime et al. (2003) |
| *Amauroderma flabellatum* | Aime et al. (2007) |
| *Bannoa tropicalis* | Parra and Aime (2019) |
| *Beauveria blattidicola* | Kepler et al. (2017) |
| **Binderoboletus* *segoi* | Henkel et al. (2016) |
| *Boletellus dicymbophilus* | Fulgenzi et al. (2008) |
| *Boletellus exiguus* | Fulgenzi et al. (2008) |
| *Boletellus piakaii* | Mayor et al. (2008) |
| *Calliderma caeruleosplendens* (now *Entoloma caeruleosplendens*) | Aime et al. (2010) |
| *Cantharellus pleurotoides* (now *Craterellus pleurotoides*) | Henkel et al. (2006a) |
| **Castellanea pakaraimophila* | Smith et al. (2015) |
| *Ceriporia subspissa* | Aime et al. (2007) |
| *Clavulina caespitosa* | Henkel et al. (2005b) |
| *Clavulina cerebriformis* | Uehling et al. (2012) |
| *Clavulina cinereoglebosa* | Uehling et al. (2012) |
| *Clavulina craterelloides* | Thacker & Henkel (2004) |
| *Clavulina dicymbetorum* | Henkel et al. (2005b) |
| *Clavulina effusa* | Uehling et al. (2012) |
| *Clavulina griseohumicola* | Henkel et al. (2005b) |
| *Clavulina guyanensis* | Wilson et al. (2012) |
| *Clavulina humicola* | Henkel et al. (2005b) |
| *Clavulina kunmudlutsa* | Henkel et al. (2011) |
| *Clavulina monodiminutiva* | Henkel et al. (2005b) |
| *Clavulina nigricans* | Thacker & Henkel (2004) |
| *Clavulina pakaraimensis* | Wilson et al. (2012) |
| *Clavulina rosiramea* | Wilson et al. (2012) |
| *Clavulina tepurumenga* | Henkel et al. (2011) |
| *Coltricia fibrosa* | Aime et al. (2007) |
| *Coltricia verrucata* | Aime et al. (2003) |
| *Coltriciella navispora* (now *Coltricia navispora*) | Aime et al. (2003) |
| *Cortinarius altissimus* | Harrower et al. (2015) |
| *Cortinarius andrewii* | Siegel et al. (2024) |
| *Cortinarius magniviscidus* | Siegel et al. (2024) |
| *Cortinarius parviviscidus* | Siegel et al. (2024) |
| **Costatisporus cyanescens* | Smith et al. (2015) |
| *Craterellus atratoides* | Wilson et al. (2012) |
| *Craterellus cinereofimbriatus* | Henkel et al. (2014b) |
| *Craterellus excelsus* | Henkel et al. (2009) |
| *Craterellus olivaceoluteus* | Henkel et al. (2014b) |
| *Craterellus strigosus* | Wilson et al. (2012) |
| *Dactylosporina cephalocystidiata* (now *Oudemansiella cephalocystidiata*) | Petersen & Hughes (2010) |
| *Dichomitus grandisporus* | Aime et al. (2007) |
| *Elaphomyces adamizans* | Castellano et al. (2016) |
| *Elaphomyces compleximurus* | Castellano et al. (2012) |
| *Elaphomyces digitatus* | Castellano et al. (2012) |
| *Entoloma fragilum* (now *Entoloma fragosum*) | Largent et al. (2008b) |
| *Entoloma illinitum* | Largent et al. (2008b) |
| *Entoloma olivaceocoloratum* | Largent et al. (2008b) |
| *Entoloma rugosostriatum* | Largent et al. (2008b) |
| *Fistulinella cinereoalba* | Fulgenzi et al. (2010) |
| *Fomitopsis minuta* | Aime et al. (2017) |
| *Fusarium xyrophilum* | Laraba et al. (2020a) |
| **Guyanagarika anomala* | Sánchez-García et al. (2016) |
| *Guyanagarika aurantia* | Sánchez-García et al. (2016) |
| *Guyanagarika pakaraimensis* | Sánchez-García et al. (2016) |
| **Guyanagaster lucianii* | Koch et al. (2017) |
| *Guyanagaster necrorhizus* | Henkel et al. (2010c) |
| **Guyanaporus* *albipodus* | Henkel et al. (2016) |
| *Inocybe ayangannae* | Matheny et al. (2003) |
| *Inocybe enigmatica* | Matheny et al. (2012) |
| *Inocybe epidendron* | Matheny et al. (2003) |
| *Inocybe lepidotella* (now *Pseudosperma lepidotellum*) | Matheny et al. (2012) |
| *Inocybe lilacinosquamosa* | Matheny et al. (2003) |
| *Inocybe magnifolia* | Matheny et al. (2012) |
| *Inocybe marginata* | Matheny et al. (2012) |
| *Inocybe pulchella* | Matheny et al. (2003) |
| *Inocybe rhodella* | Matheny et al. (2012) |
| **Jimtrappea guyanensis* | Smith et al. (2015) |
| *Lactarius aurantiolamellatus* | Nuytinck et al. (2023) |
| *Lactarius brunellus* (now *Lactifluus brunellus*) | Miller et al. (2002) |
| *Lactarius dicymbophilus* | Nuytinck et al. (2023) |
| *Lactarius guyanensis* | Nuytinck et al. (2023) |
| *Lactarius humiphilus* | Nuytinck et al. (2023) |
| *Lactarius multiceps* (now *Lactifluus multiceps*) | Miller et al. (2002) |
| *Lactarius mycenoides* | Nuytinck et al. (2023) |
| *Lactifluus subiculatus* | Miller et al. (2002) |
| *Malupa pakaraimensis* | Hernández et al. (2005) |
| *Marasmius neocrinis-equi* | Koch et al. (2020) |
| *Marasmius nidus-avis* | Koch et al. (2020) |
| *Megacollybia fusca* | Hughes et al. (2007) |
| **Meredithblackwellia* *eburnea* | Toome et al. (2013) |
| *Nolanea alboproxima* (now *Entoloma alboproximum*) | Henkel et al. (2014a) |
| *Nolanea applanata* (now *Entoloma dilutum*) | Henkel et al. (2014a) |
| *Nolanea clavata* (now *Entoloma clavatum*) | Henkel et al. (2014a) |
| *Nolanea claviformis* (now *Entoloma claviforme*) | Henkel et al. (2014a) |
| *Nolanea concentrica* (now *Entoloma concentricum*) | Henkel et al. (2014a) |
| *Nolanea furcata* (now *Entoloma furcatum*) | Henkel et al. (2014a) |
| *Nolanea mimiae* (now *Entoloma mimiae*) | Henkel et al. (2014a) |
| *Nolanea rava* (now *Entoloma ravum*) | Henkel et al. (2014a) |
| *Nolanea sinuolata* (now *Entoloma sinuolatum*) | Henkel et al. (2014a) |
| *Nolanea subsulcata* (now *Entoloma subsulcatum*) | Henkel et al. (2014a) |
| *Paraeccilia unicolorata* (now *Entoloma unicoloratum*) | Aime et al. (2010) |
| *Phlegmacium purpureofirmum* | Siegel et al. (2024) |
| *Phlegmacium purpureosolidum* | Siegel et al. (2024) |
| *Phylloporus colligatus* | Neves et al. (2010) |
| **Pseudotulostoma volvatum* | Miller Jr et al. (2001) |
| *Rhodocybe pruinosistipitata* | Henkel et al. (2010b) |
| *Rhodocybe spongiosa* | Henkel et al. (2010b) |
| *Russula gelatinivelata* | Miller et al. (2012) |
| *Russula myrmecobroma* | Miller et al. (2012) |
| *Russula paxilliformis* | Miller et al. (2012) |
| *Sarcodon pakaraimensis* (now *Neosarcodon pakaraimensis*) | Grupe et al. (2015) |
| **Singerocomus rubriflavus* | Henkel et al. (2016) |
| *Trichopilus fasciculatus* (now *Entoloma guyanense*) | Aime et al. (2010) |
| *Trichopilus luteolamellatus* (now *Entoloma luteolamellatum*) | Aime et al. (2010) |
| *Trichopilus tibiiformis* (now *Entoloma tibiiforme*) | Aime et al. (2010) |
| *Trichopilus vividus* (now *Entoloma vividum*) | Aime et al. (2010) |
| *Tylopilus exiguus* | Henkel (1999) |
| *Tylopilus orsonianus* | Fulgenzi et al. (2007) |
| *Tylopilus pakaraimensis* | Henkel (2001) |
| *Tylopilus rufonigricans* | Henkel (1999) |
| *Uredo baruensis* | Hernández et al. (2005) |
| *Uromyces neotropicalis* | Hernández et al. (2005) |
| *Wrightoporia micropora* (now *Wrightoporia microporella*) | Aime et al. (2007) |
| *Xerocomus cyaneibrunnescens* | Husbands et al. (2013) |
| *Xerocomus parvogracilis* | Husbands et al. (2013) |
| *Xerocomus potaroensis* | Husbands et al. (2013) |
| *Xylaria karyophthora* | Husbands et al. (2018) |
