# Supplementary material for: The contribution of tropical long-term studies to mycology
Source: IMA Fungus. 2024 Nov 11;15:35. doi: 10.1186/s43008-024-00166-5 (PMC11552369; doi:10.1186/s43008-024-00166-5)
Supplement: Supplementary file 14 — Additional file 14. Table S2. Papers published with data from long-term studies in Guyana. [file 43008_2024_166_MOESM14_ESM.docx]

Table S2. Papers published with data from long-term studies in Guyana and whether the paper broadly addresses alpha diversity or other topics.

| **Reference** | **Alpha Diversity** | **Other Diversity (ecological, genetic, etc.)** |
| --- | --- | --- |
| Aime MC, Brearley FQ (2012) Tropical fungal diversity: closing the gap between species estimates and species discovery. Biodiversity and Conservation 21:2177–2180. doi:10.1007/s10531-012-0338-7 |  | x |
| Aime MC, Henkel TW, Ryvarden L (2003) Studies in neotropical polypores 15: new and interesting species from Guyana. Mycologia 95:614–619. doi:10.1080/15572536.2004.11833065 | x |  |
| Aime MC, Largent DL, Henkel TW, Baroni TJ (2010) The Entolomataceae of the Pakaraima Mountains of Guyana IV: new species of *Calliderma*, *Paraeccilia* and *Trichopilus*. Mycologia 102:633–649. doi:10.3852/09-162 | x |  |
| Aime MC, Phillips-Mora W (2005) The causal agents of witches’ broom and frosty pod rot of cacao (chocolate, *Theobroma cacao*) form a new lineage of Marasmiaceae. Mycologia 97(5):1012–1022. doi:10.3852/mycologia.97.5.1012 | x |  |
| Aime MC, Ryvarden L, Henkel TW (2007) Studies in Neotropical polypores 22: Additional new and rare species from Guyana. Synopsis Fungorum 23:15–31. | x |  |
| Castellano MA, Dentinger B, Séné O, Elliott TF, Truong C, Henkel TW (2016) New species of *Elaphomyces* (Elaphomycetaceae, Eurotiales, Ascomycota) from tropical rainforests of Cameroon and Guyana. IMA Fungus 7:59–73. doi:10.5598/imafungus.2016.07.01.05 | x |  |
| Castellano MA, Henkel TW, Miller SL, Smith ME, Aime MC (2012) New *Elaphomyces* species (Elaphomycetaceae, Eurotiales, Ascomycota) from Guyana. Mycologia 104(5):1244–1249. doi:10.3852/12-061 | x |  |
| Delevich C, Koch RA, Dentinger BTM, Aime MC, Henkel TW (2021) Ectomycorrhizal fungal community assembly on seedlings of a Neotropical monodominant tree. Biotropica 53:1486-1497. doi:10.1111/btp.12989 |  | x |
| Dentinger BTM, Gaya E, O'Brien H, Suz LM, Lachlan R, Diaz-Valderrama JR, Koch RA, Aime MC (2016) Tales from the crypt: genome mining from fungarium specimens improves resolution of the mushroom tree of life. Biological Journal of the Linnean Society 117:11–32. doi:10.1111/bij.12553 |  | x |
| Fulgenzi TD, Halling RE, Henkel TW (2010) *Fistulinella cinereoalba* sp. nov. and new distribution records for *Austroboletus* from Guyana. Mycologia 102(1):224–232. doi:10.3852/09-059 | x |  |
| Fulgenzi TD, Henkel TW, Halling RE (2007) *Tylopilus orsonianus* sp. nov. and *Tylopilus eximius* from Guyana. Mycologia 99(4):622–627. doi:10.3852/mycologia.99.4.622 | x |  |
| Fulgenzi TD, Mayor JR, Henkel TW, Halling RE (2008) New species of *Boletellus* from Guyana. Mycologia 100(3):490–495. doi:10.3852/07-170R | x |  |
| Grupe AC, Baker AD, Uehling JK, Smith ME, Baroni TJ, Lodge DJ, Henkel TW (2015) *Sarcodon* in the Neotropics I: new species from Guyana, Puerto Rico and Belize. Mycologia 107(3):591–606. doi:10.3852/14-185 | x |  |
| Haelewaters D, Stallman JK, Henkel TW, Aime MC (2022) Molecular phylogenetic analyses and micromorphology reveal placement of the enigmatic tropical discomycete *Polydiscidium* in *Sclerococcum* (Sclerococcales, Eurotiomycetes). Mycologia 114(3):626–641. doi:10.1080/00275514.2022.2048625 | x |  |
| Harrower E, Bougher NL, Winterbottom C, Henkel TW, Horak E, Matheny PB (2015) New species in *Cortinarius* section *Cortinarius* (Agaricales) from the Americas and Australasia. MycoKeys 11:1-21. doi:10.3897/mycokeys.11.5409 | x |  |
| Henkel TW (1999) New taxa and distribution records of *Tylopilus* from *Dicymbe* forests of Guyana. Mycologia 91(4):655–665. doi:10.1080/00275514.1999.12061065 | x |  |
| Henkel TW (2001) *Tylopilus pakaraimensis* sp. nov., a new bolete in *Tylopilus* section Potamogetones from Guyana. Mycotaxon 78:105–114. | x |  |
| Henkel TW (2003) Monodominance in the ectomycorrhizal *Dicymbe corymbosa* (Caesalpiniaceae) from Guyana. Journal of Tropical Ecology 19(4):417–437. doi:10.1017/S0266467403003468 |  | x |
| Henkel TW (2004) Manufacturing procedures and microbiological aspects of Parakari, a novel fermented beverage of the wapisiana amerindians of Guyana. Economic Botany 58:25–37. doi:10.1663/0013-0001(2004)058[0025:MPAMAO]2.0.CO;2 |  | x |
| Henkel TW, Aime MC, Chin M, Andrew C (2004b) Edible mushrooms from Guyana. Mycologist 18:104–111. | x | x |
| Henkel TW, Aime MC, Chin MM, Miller SL, Vilgalys R, Smith ME (2012) Ectomycorrhizal fungal sporocarp diversity and discovery of new taxa in *Dicymbe* monodominant forests of the Guiana Shield. Biodiversity and Conservation 21:2195–2220. doi:10.1007/s10531-011-0166-1 | x | x |
| Henkel TW, Aime MC, Largent DL, Baroni TJ (2010a) The Entolomataceae of the Pakaraima Mountains of Guyana V: new species of *Alboleptonia*. Mycotaxon 114(1):115–126. doi:10.5248/114.115 | x |  |
| Henkel TW, Aime MC, Largent DL, Baroni TJ (2014a) The Entolomataceae of the Pakaraima Mountains of Guyana 6: ten new species and a new combination in *Nolanea*. Mycotaxon 129(1):119–148. doi:10.5248/129.119 | x |  |
| Henkel TW, Aime M, Mehl H (2009) *Craterellus excelsus* sp. nov. from Guyana. Mycotaxon 107(1):201–208. | x |  |
| Henkel TW, Aime MC, Mehl H, Miller SL (2006a) *Cantharellus pleurotoides*, a new and unusual basidiomycete from Guyana. Mycological Research 110(12):1409–1412. doi:10.1016/j.mycres.2006.09.010 | x |  |
| Henkel TW, Aime MC, Miller SL (2000) Systematics of pleurotoid Russulaceae from Guyana and Japan, with notes on their ectomycorrhizal status. Mycologia 92(6):1119–1132. doi:10.1080/00275514.2000.12061259 | x | x |
| Henkel TW, Aime MC, Uehling JK, Smith ME (2011) New species and distribution records for *Clavulina* (Cantharellales, Basidiomycota) from the Guiana Shield. Mycologia 103(4):883–894. doi:10.3852/10-355 | x |  |
| Henkel TW, James TY, Miller SL, Aime MC, Miller OK (2006b) The mycorrhizal status of *Pseudotulostoma volvata* (Elaphomycetaceae, Eurotiales, Ascomycota). Mycorrhiza 16:241–244. doi:10.1007/s00572-006-0040-2 |  | x |
| Henkel TW, Largent DL, Aime MC, Baroni TJ (2010b). The Entolomataceae of the Pakaraima Mountains of Guyana III: new species of *Rhodocybe*. Mycoscience 51(1):23–27. doi:10.1007/S10267-009-0008-0 | x |  |
| Henkel TW, Mayor JR, Woolley LP (2005a) Mast fruiting and seedling survival of the ectomycorrhizal, monodominant *Dicymbe corymbosa* (Caesalpiniaceae) in Guyana. New Phytologist 167:543–556. doi:10.1111/j.1469-8137.2005.01431.x |  | x |
| Henkel TW, Meszaros R, Aime MC, Kennedy A (2005b) New *Clavulina* species from the Pakaraima mountains of Guyana. Mycological Progress 4:343–350. doi:10.1007/s11557-006-0140-6 | x |  |
| Henkel TW, Obase K, Husbands D, Uehling JK, Bonito G, Aime MC, Smith ME (2016) New Boletaceae taxa from Guyana: *Binderoboletus segoi* gen. and sp. nov., *Guyanaporus albipodus* gen. and sp. nov., *Singerocomus rubriflavus* gen. and sp. nov., and a new combination for *Xerocomus inundabilis*. Mycologia 108:157–173. doi:10.3852/15-075 | x |  |
| Henkel TW, Roberts P, Aime MC (2004a) Sebacinoid species from the Pakaraima Mountains of Guyana. Mycotaxon 89:433–439. | x |  |
| Henkel TW, Smith ME, Aime MC (2010c) *Guyanagaster*, a new wood-decaying sequestrate fungal genus related to Armillaria (Physalacriaceae, Agaricales, Basidiomycota). American Journal of Botany 97(9):1474–1484. doi:10.3732/ajb.1000097 | x | x |
| Henkel TW, Terborgh J, Vilgalys RJ (2002) Ectomycorrhizal fungi and their leguminous hosts in the Pakaraima Mountains of Guyana. Mycological Research 106(5):515–531. doi:10.1017/S0953756202005919 | x | x |
| Henkel TW, Wilson AW, Aime MC, Dierks J, Uehling JK, Roy M, Schimann H, Wartchow F, Mueller GM (2014b) Cantharellaceae of Guyana II: New species of *Craterellus*, new South American distribution records for *Cantharellus guyanensis* and *Craterellus excelsus*, and a key to the Neotropical taxa. Mycologia 106(2):307–324. doi:10.3852/106.2.307 | x |  |
| Hernández JR, Aime M, Henkel TW (2005) The rust fungi (Uredinales) of Guyana. Sydowia 57(2):189–222. | x | x |
| Hughes KW, Petersen RH, Mata JL, Psurtseva NV, Kovalenko AE, Morozova OV, Lickey EB, Cifuentes Blanco J, Lewis DP, Nagasawa E, Halling RE, Takehashi S, Aime MC, Bau T, Henkel T (2007) *Megacollybia* (Agaricales). Report of the Tottori Mycological Institute 45:1–57. | x |  |
| Husbands DR, Aime MC (2018) Emerging forest diseases: A case study of Greenheart (*Chlorocardium* spp., Lauraceae) and the newly described fungus, *Xylaria karyophthora*. Forests 9(6):365. doi:10.3390/f9060365 |  | x |
| Husbands DR, Henkel TW, Bonito G, Vilgalys R, Smith ME (2013) New species of *Xerocomus* (Boletales) from the Guiana Shield, with notes on their mycorrhizal status and fruiting occurrence. Mycologia 105(2):422–435. doi:10.3852/12-146 | x |  |
| Husbands DR, Urbina H, Lewis SM, Aime MC (2018) *Xylaria karyophthora*: a new seed-inhabiting fungus of Greenheart from Guyana. Mycologia 110(2):434–447. doi:10.1080/00275514.2018.1457349 | x | x |
| Kepler RM, Luangsa-ard JJ, Hywel-Jones NL, Quandt CA, Sung GH, Rehner SA, Aime MC, Henkel TW, Sanjuan T, Zare R, Chen M, Li Z, Rossman AY, Spatafora JW, Shrestha B (2017) A phylogenetically-based nomenclature for Cordycipitaceae (Hypocreales). IMA Fungus 8: 335-353. doi:10.5598/imafungus.2017.08.02.08 | x |  |
| Koch RA, Aime MC (2018) Population structure of *Guyanagaster necrorhizus* supports termite dispersal for this enigmatic fungus. Molecular Ecology 27(12):2667–2679. doi:10.1111/mec.14710 |  | x |
| Koch RA, Bach CE, Pirro, S Aime MC (2022) Draft genome of an unusual ectomycorrhizal fungus, P*seudotulostoma volvatum*. Microbiology Resource Announcements 11: e00801-21. doi:10.1128/mra.00801-21 |  | x |
| Koch RA, Brann M, Aime MC (2022) Viability of fungal rhizomorphs used in bird nest construction in tropical rainforests. Symbiosis 87:175–179. doi:10.1007/s13199-022-00856-x |  | x |
| Koch RA, Liu J, Brann M, Jumbam B, Siegel N, Aime MC (2020) Marasmioid rhizomorphs in bird nests: Species diversity, functional specificity, and new species from the tropics. Mycologia 112(6):1086–1103. doi:10.1080/00275514.2020.1788892 | x | x |
| Koch RA, Lodge DJ, Sourell S, Nakasone K, McCoy AG, Aime MC (2018) Tying up loose threads: revised taxonomy and phylogeny of an avian-dispersed neotropical rhizomorph-forming fungus. Mycological Progress 17:989–998. doi:10.1007/s11557-018-1411-8 | x |  |
| Koch RA, Wilson AW, Séné O, Henkel TW, Aime MC (2017) Resolved phylogeny and biogeography of the root pathogen *Armillaria* and its gasteroid relative, *Guyanagaster*. BMC Evolutionary Biology 17:33. doi:10.1186/s12862-017-0877-3 |  | x |
| Koch RA, Yoon GM, Aryal UK, Lail K, Amirebrahimi M, LaButti K, Lipzen A, Riley R, Barry K, Henrissat B, Grigoriev IV, Herr JR, Aime MC (2021) Symbiotic nitrogen fixation in the reproductive structures of a basidiomycete fungus. Current Biology 31:3905-3914.e6. doi:10.1016/j.cub.2021.06.033 |  | x |
| Lambert C, Shao L, Zenga H, Surup F, Saetang P, Aime MC, Husbands DR, Rottner K, Stradal TEB, Stadler M. 2023. Cytochalasans produced by *Xylaria karyophthora* and their biological activities. Mycologia 115(3): 277–287. doi:10.1080/00275514.2023.2188868 |  | x |
| Laraba I, Kim H-S, Proctor RH, Busman M, O’Donnell K, Felker FC, Aime MC, Koch RA, Wurdack KJ (2020a) *Fusarium xyrophilum*, sp. nov., a member of the *Fusarium fujikuroi* species complex recovered from pseudoflowers on yellow-eyed grass (*Xyris* spp.) from Guyana. Mycologia 112(1):39–51. doi:10.1080/00275514.2019.1668991 | x | x |
| Laraba I, McCormick SP, Vaughan MM, Proctor RH, Busman M et al (2020b) Pseudoflowers produced by *Fusarium xyrophilum* on yellow-eyed grass (Xyris spp.) in Guyana: A novel floral mimicry system? Fungal Genetics and Biology 144:103466. doi:10.1016/j.fgb.2020.103466 |  | x |
| Largent DL, Aime MC, Henkel TW, Baroni TJ (2008a) The Entolomataceae of the Pakaraima Mountains of Guyana 2: *Inocephalus dragonosporus* comb. nov. Mycotaxon 105:185–190. | x |  |
| Largent DL, Henkel TW, Aime MC, Baroni TJ (2008b) The Entolomataceae of the Pakaraima Mountains of Guyana I: Four New Species of *Entoloma* s. str. Mycologia 100(1):132–140. doi:10.1080/15572536.2008.11832505 | x |  |
| Lodge D, Læssøe T, Aime MC, Henkel TW (2008) Montane and cloud forest specialists among neotropical *Xylaria* species. North American Fungi 3:193–213. doi:10.2509/naf2008.003.00713 | x | x |
| Lodge DJ, Padamsee M, Matheny PB, Aime MC, Cantrell SA, Boertmann D, Kovalenko A, Vizzini A, Dentinger BTM, Kirk PM, Ainsworth AM, Moncalvo J-M, Vilgalys R, Larsson E, Lücking R, Griffith GW, Smith ME, Norvell LL, Desjardin DE, Redhead SA, Ovrebo CL, Lickey EB, Ercole E, Hughes KW, Courtecuisse R, Young A, Binder M, Minnis AM, Lindner DL, Ortiz-Santana B, Haight J, Læssøe T, Baroni TJ, Geml J, Hattori T (2014) Molecular phylogeny, morphology, pigment chemistry and ecology in Hygrophoraceae (Agaricales). Fungal Diversity 64:1–99. doi:10.1007/s13225-013-0259-0 | x | x |
| Magnago AC, Alves-Silva G, Henkel TW, da Silveira RMB (2022) New genera, species, and combinations of Boletaceae from Brazil and Guyana. Mycologia 114:607–625. doi:10.1080/00275514.2022.2037307 | x |  |
| Matheny PB, Aime MC, Bougher N, Buyck B, Desjardin D, Horak E, Kropp B, Lodge DJ, Soytong K, Trappe JM, Hibbett DS (2009) Out of the Paleotropics? Historical biogeography and diversification of the cosmopolitan ectomycorrhizal mushroom family Inocybaceae. Journal of Biogeography 36(4):577–592. doi:10.1111/j.1365-2699.2008.02055.x |  | x |
| Matheny PB, Aime MC, Henkel TW (2003) New species of *Inocybe* from *Dicymbe* forests of Guyana. Mycological Research 107(4):495–505. doi:10.1017/s0953756203007627 | x |  |
| Matheny P, Aime M, Smith M, Henkel TW (2012) New species and reports of *Inocybe* (Agaricales) from Guyana. Kurtziana 37(1):23–39. | x |  |
| Mayor JR, Fulgenzi TD, Henkel TW, Halling RE (2008) *Boletellus piakaii* sp. nov. and a new distribution record for *Boletellus ananas* var. *ananas* from Guyana. Mycotaxon 105:387–398. | x |  |
| Mayor JR, Henkel TW (2006) Do ectomycorrhizas alter leaf-litter decomposition in monodominant tropical forests of Guyana? New Phytologist 169(3):579–588. doi:10.1111/j.1469-8137.2005.01607.x |  | x |
| Mighell KS, Henkel TW, Koch RA, Goss A, Aime MC (2019) New species of *Amanita* subgen. *Lepidella* from Guyana. Fungal Systematics and Evolution 3:1–12. doi:10.3114%2Ffuse.2019.03.01 | x |  |
| Miller OK, Henkel TW, James TY, Miller SL (2001) *Pseudotulostoma*, a remarkable new volvate genus in the Elaphomycetaceae from Guyana. Mycological Research 105(10):1268–1272. doi:10.1017/S095375620100466X | x |  |
| Miller SL, Aime MC, Henkel TW (2002) Russulaceae of the Pakaraima Mountains of Guyana. I. New species of pleurotoid *Lactarius*. Mycologia 94(3):545–553. doi:10.1080/15572536.2003.11833220 | x |  |
| Miller SL, Aime MC, Henkel TW (2012) Russulaceae of the Pakaraima mountains of Guyana 2. New species of *Russula* and *Lactifluus*. Mycotaxon 121(1):233–253. doi:10.5248/121.233 | x |  |
| Miller SL, Henkel TW (2004) Biology and molecular ecology of subiculate *Lactarius* species from Guyana. Memoirs of the New York Botanical Garden 89:297–314. | x | x |
| Neves M, Henkel TW, Halling R (2010) *Phylloporus colligatus* sp. nov., a new gilled bolete from Guyana. Mycotaxon 111(1):143–148. doi:10.5248/111.143 | x |  |
| Nuytinck J, Henkel TW, Delgat L, Milisav K, Noordermeer C, Verbeken A, Aime MC (2023) Russulaceae of the Pakaraima Mountains of Guyana. IV. New species forming a distinct lineage of *Lactarius* subg. *Plinthogalus*. Mycologia 115(1):69–86. doi:10.1080/00275514.2022.2125712 | x |  |
| Okane I, Ono Y, Ohmachi K, Aime MC, Yamaoka Y (2021) Phylogenetic relationships among fern rust fungi and *Desmella lygodii* comb. nov. Mycoscience 62(6): 364-372. doi:10.47371%2Fmycosci.2021.06.006 | x |  |
| Parra PP, Aime MC (2019) New species of *Bannoa* described from the tropics and the first report of the genus in South America. Mycologia 111(6):953–964. doi:10.1080/00275514.2019.1647397 | x |  |
| van de Peppel LJJ, Aime MC, Læssøe T, Pedersen OS, Coimbra VRM, Kuyper TW, Stubbe D, Aanen DK, Baroni TH (2022) Four new genera and six new species of lyophylloid agarics (Agaricales, Basidiomycota) from three different continents. Mycological progress 21:85. doi:10.1007/s11557-022-01836-7 | x |  |
| Petersen RH, Hughes KW (2010) The *Xerula*/*Oudemansiella* Complex (Agaricales). Beihefte zu Nova Hedw vol. 137; J. Cramer, Stuttgart. | x |  |
| Sánchez-García M, Henkel TW, Aime MC, Smith ME, Matheny PB (2016) *Guyanagarika*, a new ectomycorrhizal genus of Agaricales from the neotropics. Fungal Biology 120(12):1540–1553. doi:10.1016/j.funbio.2016.08.005 | x |  |
| Siegel N, Henkel TW, Adams S, Cooper J, Aime MC (2024) New Cortinariaceae species associated with *Dicymbe*, *Aldina*, and *Pakaraimaea* in Guyana. Mycologia 1–17. doi:10.1080/00275514.2024.2367399 | x |  |
| Simmons C, Henkel TW, Bas C (2002) The genus *Amanita* in the Pakaraima mountains of Guyana. Persoonia 17(4):563–582. | x |  |
| Smith ME, Amses K, Elliott T, Aime MC, Henkel TW (2015) New sequestrate fungi from Guyana: *Jimtrappea guyanensis* gen. sp. nov., *Castellanea pakaraimophila* gen. sp. nov., and *Costatisporus cyanescens* gen. sp. nov. (Boletaceae, Boletales). IMA Fungus 6(2):263–283. doi:10.5598/imafungus.2015.06.02.03 | x |  |
| Smith ME, Henkel TW, Aime MC, Fremier AK, Vilgalys R (2011) Ectomycorrhizal fungal diversity and community structure on three co-occurring leguminous canopy tree species in a Neotropical rainforest. New Phytologist 192:699–712. doi:10.1111/j.1469-8137.2011.03844.x |  | x |
| Smith ME, Henkel TW, Uehling JK, Fremier AK, Clarke HD, Vilgalys R (2013) The ectomycorrhizal fungal community in a neotropical forest dominated by the endemic dipterocarp *Pakaraimaea dipterocarpacea*. PLoS One 8:e55160. doi:10.1371/journal.pone.0055160 |  | x |
| Smith ME, Henkel TW, Williams GC, Aime MC, Fremier AK, Vilgalys R (2017) Investigating niche partitioning of ectomycorrhizal fungi in specialized rooting zones of the monodominant leguminous tree *Dicymbe corymbosa*. New Phytologist 215:443–453. doi:10.1111/nph.14570 |  | x |
| Thacker JR, Henkel TW (2004) New species of *Clavulina* from Guyana. Mycologia 96(3):650–657. doi:10.1080/15572536.2005.11832961 | x |  |
| Toome M, Roberson R, Aime MC (2013) *Meredithblackwellia eburnea* gen. et sp. nov., Kriegeriaceae fam. nov. and Kriegeriales ord. nov—toward resolving higher-level classification in Microbotryomycetes. Mycologia 105(2):486–495. doi:10.3852/12-251 | x | x |
| Uehling JK, Henkel TW, Aime MC, Vilgalys R, Smith ME (2012a) New species and distribution records for *Clavulina* (Cantharellales, Basidiomycota) from the Guiana Shield, with a key to the lowland neotropical taxa. Fungal Biology 116(12):1263–1274. doi:10.1016/j.funbio.2012.09.004 | x |  |
| Uehling JK, Henkel TW, Aime MC, Vilgalys R, Smith ME (2012b) New species of *Clavulina* (Cantharellales, Basidiomycota) with resupinate and effused basidiomata from the Guiana Shield. Mycologia 104(2):547–556. doi:10.3852/11-130 | x |  |
| Wilson AW, Aime MC, Dierks J, Mueller GM, Henkel TW (2012) Cantharellaceae of Guyana I: new species, combinations and distribution records of *Craterellus* and a synopsis of known taxa. Mycologia 104(6):1466–1477. doi:10.3852/11-412 | x |  |
