# Supplementary material for: The contribution of tropical long-term studies to mycology
Source: IMA Fungus. 2024 Nov 11;15:35. doi: 10.1186/s43008-024-00166-5 (PMC11552369; doi:10.1186/s43008-024-00166-5)
Supplement: Supplementary file 15 — Additional file 15. Table S3. New species and genera originating from long-term studies in Cameroon. [file 43008_2024_166_MOESM15_ESM.docx]

Table S3. New species and genera originating from long-term studies in Cameroon. Asterisks denote newly described genera.

| **Name** | **Reference** |
| --- | --- |
| *Amanita goossensfontanae* | Mighell KS, Henkel TW, Koch RA, Chin ML, Brann MA Aime MC (2021) *Amanita* in the Guineo-Congolian rainforest: Epitypes and new species from the Dja Biosphere Reserve, Cameroon. Mycologia 113(1):168-190. doi:10.1080/00275514.2020.1816386 |
| *Amanita luteolamellata* | Mighell KS, Henkel TW, Koch RA, Chin ML, Brann MA Aime MC (2021) *Amanita* in the Guineo-Congolian rainforest: Epitypes and new species from the Dja Biosphere Reserve, Cameroon. Mycologia 113(1):168-190. doi:10.1080/00275514.2020.1816386 |
| *Amanita minima* | Mighell KS, Henkel TW, Koch RA, Chin ML, Brann MA Aime MC (2021) *Amanita* in the Guineo-Congolian rainforest: Epitypes and new species from the Dja Biosphere Reserve, Cameroon. Mycologia 113(1):168-190. doi:10.1080/00275514.2020.1816386 |
| *Auritella hispida* | Matheny PB, Henkel TW, Séné O, Korotkin HB, Dentinger BTM, Aime MC (2017) New species of *Auritella* (Inocybaceae) from Cameroon, with a worldwide key to the known species. IMA Fungus 8:287–298. doi:10.5598/imafungus.2017.08.02.06 |
| *Auritella spiculosa* | Matheny PB, Henkel TW, Séné O, Korotkin HB, Dentinger BTM, Aime MC (2017) New species of *Auritella* (Inocybaceae) from Cameroon, with a worldwide key to the known species. IMA Fungus 8:287–298. doi:10.5598/imafungus.2017.08.02.06 |
| *Cantharellus albidosquamosus* | Rossi R, Das K, Hembrom ME, Santamaria S, Parihar A, Ghosh A, Henkel TW, Hofstetter V, Randrianjohany É, Vizzini A, Wang X-H, Buyck B (2020) Fungal Biodiversity Profiles 91-100. Cryptogamie Mycologie 41(4):69–107. doi:10.5252/cryptogamie-mycologie2020v41a4 |
| *Elaphomyces favosus* | Castellano MA, Dentinger B, Séné O, Elliott TF, Truong C, Henkel TW (2016) New species of *Elaphomyces* (Elaphomycetaceae, Eurotiales, Ascomycota) from tropical rainforests of Cameroon and Guyana. IMA Fungus 7:59–73. doi:10.5598/imafungus.2016.07.01.05 |
| *Elaphomyces iuppitercellus* | Castellano MA, Dentinger B, Séné O, Elliott TF, Truong C, Henkel TW (2016) New species of *Elaphomyces* (Elaphomycetaceae, Eurotiales, Ascomycota) from tropical rainforests of Cameroon and Guyana. IMA Fungus 7:59–73. doi:10.5598/imafungus.2016.07.01.05 |
| *Elaphomyces labyrinthinus* | Castellano MA, Dentinger B, Séné O, Elliott TF, Truong C, Henkel TW (2016) New species of *Elaphomyces* (Elaphomycetaceae, Eurotiales, Ascomycota) from tropical rainforests of Cameroon and Guyana. IMA Fungus 7:59–73. doi:10.5598/imafungus.2016.07.01.05 |
| *Entoloma bisterigmatum* | Largent DL, Henkel TW, Siegel N, Koch RA, Séné O, Hageman KM, Aime MC (2020) New species of Entolomataceae from Cameroon. Fungal Syst Evol. 5:151–167. doi:10.3114/fuse.2020.05.10 |
| *Entoloma brunneoloaurantiacum* | Largent DL, Henkel TW, Siegel N, Koch RA, Séné O, Hageman KM, Aime MC (2020) New species of Entolomataceae from Cameroon. Fungal Syst Evol. 5:151–167. doi:10.3114/fuse.2020.05.10 |
| *Entoloma djaense* | Largent DL, Henkel TW, Siegel N, Koch RA, Séné O, Hageman KM, Aime MC (2020) New species of Entolomataceae from Cameroon. Fungal Syst Evol. 5:151–167. doi:10.3114/fuse.2020.05.10 |
| *Entoloma intricatum* | Largent DL, Henkel TW, Siegel N, Koch RA, Séné O, Hageman KM, Aime MC (2020) New species of Entolomataceae from Cameroon. Fungal Syst Evol. 5:151–167. doi:10.3114/fuse.2020.05.10 |
| *Entoloma parvistellatum* | Largent DL, Henkel TW, Siegel N, Koch RA, Séné O, Hageman KM, Aime MC (2020) New species of Entolomataceae from Cameroon. Fungal Syst Evol. 5:151–167. doi:10.3114/fuse.2020.05.10 |
| *Entoloma versiforme* | Largent DL, Henkel TW, Siegel N, Koch RA, Séné O, Hageman KM, Aime MC (2020) New species of Entolomataceae from Cameroon. Fungal Syst Evol. 5:151–167. doi:10.3114/fuse.2020.05.10 |
| *Hericium bembedajense* | Jumbam B, Haelewaters D, Koch RA, Dentinger BTM, Henkel TW, Aime MC (2019) A new and unusual species of *Hericium* (Basidiomycota: Russulales, Hericiaceae) from the Dja Biosphere Reserve, Cameroon. Mycol Progress 18:1253–1262. doi:10.1007/s11557-019-01530-1 |
| **Kombocles bakaiana* | Castellano MA, Elliott TF, Truong C, Séné O, Dentinger, BTM, Henkel TW (2016) *Kombocles bakaiana* gen. sp. nov. (Boletaceae), a new sequestrate fungus from Cameroon. IMA Fungus 7:239–245. doi:10.5598/imafungus.2016.07.02.03 |
| *Marasmius nidus-avis* | Koch RA, Liu J, Brann M, Jumbam B, Siegel N, Aime MC (2020) Marasmioid rhizomorphs in bird nests: Species diversity, functional specificity, and new species from the tropics. Mycologia 112(6):1086–1103. doi:10.1080/00275514.2020.1788892 |
