# Supplementary material for: The contribution of tropical long-term studies to mycology
Source: IMA Fungus. 2024 Nov 11;15:35. doi: 10.1186/s43008-024-00166-5 (PMC11552369; doi:10.1186/s43008-024-00166-5)
Supplement: Supplementary file 16 — Additional file 16. Table S4. Clavulina species described since 2000 [file 43008_2024_166_MOESM16_ESM.docx]

Table S4. *Clavulina* species described since 2000. Taxa described from the Upper Potaro River Study are in bold.

| *Clavulina* specific epithet | Reference | Notes |
| --- | --- | --- |
| *alba* | de Meiras-Ottoni A, Gibertoni TB (2023) Clavarioid fungi from Brazil: novelties in *Clavulina* (Cantharellales). Mycological Progress 22:25. doi:10.1007/s11557-023-01873-w |  |
| *alpina* | Franchi P, Marchetti M (2018) Due nuove specie italiane del Genere *Clavulina*. Rivista Di Micologia 61(1):3–21. |  |
| *arboreiparva* | Pérez-Pazos E, Villegas-Ríos M, Garibay-Orijel R, Salas-Lizana R (2019) Two new species of *Clavulina* and the first record of *Clavulina reae* from temperate *Abies religiosa* forests in central Mexico. Mycological Progress 18:1187–1200. doi:10.1007/s11557-019-01516-z |  |
| *arcuatus* | Douanla-Meli C (2007) Fungi of Cameroon: Ecological diversity with emphasis on the taxonomy of non-gilled hymenomycetes from the Mbalmayo Forest Reserve. J. Cramer, Berlin. |  |
| *baiyunensis* | Huang XX, Chen YX, Lin ZJ, Liang YS, Qiu LH (2023) Three new *Clavulina* species from Baiyun Mountain, Guangzhou, China. Mycological Progress 22(85). https://doi.org/10.1007/s11557-023-01929-x |  |
| ***caespitosa*** | Henkel TW, Meszaros R, Aime MC, Kennedy A (2005b) New *Clavulina* species from the Pakaraima mountains of Guyana. Mycological Progress 4:343–350. doi:10.1007/s11557-006-0140-6 |  |
| ***cerebriformis*** | Uehling JK, Henkel TW, Aime MC, Vilgalys R, Smith ME (2012b) New species of *Clavulina* (Cantharellales, Basidiomycota) with resupinate and effused basidiomata from the Guiana Shield. Mycologia 104(2):547–556. doi:10.3852/11-130 |  |
| ***cinereoglebosa*** | Uehling JK, Henkel TW, Aime MC, Vilgalys R, Smith ME (2012b) New species of *Clavulina* (Cantharellales, Basidiomycota) with resupinate and effused basidiomata from the Guiana Shield. Mycologia 104(2):547–556. doi:10.3852/11-130 |  |
| ***craterelloides*** | Thacker JR, Henkel TW (2004) New species of *Clavulina* from Guyana. Mycologia 96(3):650–657. doi:10.1080/15572536.2005.11832961 |  |
| *crystallifera* | de Meiras-Ottoni A, Gibertoni TB (2023) Clavarioid fungi from Brazil: novelties in *Clavulina* (Cantharellales). Mycological Progress 22:25. doi:10.1007/s11557-023-01873-w |  |
| *curva* | Liu XF, Deng PT, Yan J, Zhang P (2024) Update on the taxonomy of *Clavulina* in China I: description of three new species and one newly recorded species. Mycological Progress 23:45. doi:10.1007/s11557-024-01980-2 |  |
| *cystidiata* | de Meiras-Ottoni A, Gibertoni TB (2023) Clavarioid fungi from Brazil: novelties in *Clavulina* (Cantharellales). Mycological Progress 22:25. doi:10.1007/s11557-023-01873-w |  |
| ***dicymbetorum*** | Henkel TW, Meszaros R, Aime MC, Kennedy A (2005b) New *Clavulina* species from the Pakaraima mountains of Guyana. Mycological Progress 4:343–350. doi:10.1007/s11557-006-0140-6 |  |
| *eburnea* | Deng PT, Liu XF, Yan J, Zhang P (2024) Update on the taxonomy of *Clavulina* in China II: description of four new species from Hainan Island. Mycological Progress 23:48. doi:10.1007/s11557-024-01988-8 |  |
| ***effusa*** | Uehling JK, Henkel TW, Aime MC, Vilgalys R, Smith ME (2012b) New species of *Clavulina* (Cantharellales, Basidiomycota) with resupinate and effused basidiomata from the Guiana Shield. Mycologia 104(2):547–556. doi:10.3852/11-130 |  |
| *etruriae* | Franchi P, Marchetti M (2018) Due nuove specie italiane del Genere *Clavulina*. Rivista Di Micologia 61(1):3–21. |  |
| *flava* | Wu C-L, He Y, Yan J, Zhang P (2019) Two new species of *Clavulina* (Cantharellales) from southwestern China based on morphological and molecular evidence. Mycological Progress 18:1071–1078. doi:10.1007/s11557-019-01506-1 |  |
| *grisea* | Tibpromma S, Hyde KD, Jeewon R, Maharachchikumbura SSN, Liu J-K, Bhat DJ, Jones EBG, McKenzie EHC, Camporesi E, Bulgakov TS, Doilom M, Azevedo Santiago ALCM de, Das K, Manimohan P, Gibertoni TB, Lim YW, Ekanayaka AH, Thongbai B, Lee HB, Yang J-B, Kirk PM, Sysouphanthong P, Singh SK, Boonmee S, Dong W, Raj KNA, Latha KPD, Phookamsak R, Phukhamsakda C, et al. (2017) Fungal diversity notes 491–602: taxonomic and phylogenetic contributions to fungal taxa. Fungal Diversity 83:1–261. doi:10.1007/s13225-017-0378-0 |  |
| ***griseohumicola*** | Henkel TW, Meszaros R, Aime MC, Kennedy A (2005b) New *Clavulina* species from the Pakaraima mountains of Guyana. Mycological Progress 4:343–350. doi:10.1007/s11557-006-0140-6 |  |
| ***guyanensis*** | Uehling JK, Henkel TW, Aime MC, Vilgalys R, Smith ME (2012a) New species and distribution records for *Clavulina* (Cantharellales, Basidiomycota) from the Guiana Shield, with a key to the lowland neotropical taxa. Fungal Biology 116(12):1263–1274. doi:10.1016/j.funbio.2012.09.004 |  |
| *hainanensis* | Deng PT, Liu XF, Yan J, Zhang P (2024) Update on the taxonomy of *Clavulina* in China II: description of four new species from Hainan Island. Mycological Progress 23:48. doi:10.1007/s11557-024-01988-8 |  |
| ***humicola*** | Henkel TW, Meszaros R, Aime MC, Kennedy A (2005b) New *Clavulina* species from the Pakaraima mountains of Guyana. Mycological Progress 4:343–350. doi:10.1007/s11557-006-0140-6 |  |
| *incrustata* | Wartchow F (2012) *Clavulina incrustata*, a new species from Pernambuco, Brazil. Cryptogamie, Mycologie 33(1):105-113. doi:10.7872/crym.v33.iss1.2012.105 |  |
| *iris* | Crous PW, Wingfield MJ, Lombard L, Roets F, Swart WJ, Alvarado P, Carnegie AJ, Moreno G, Luangsaard J, Thangavel R, Alexandrova AV, Baseia IG, Bellanger J-M, Bessette AE, Bessette AR, De la Peña-Lastra S, García D, Gené J, Pham THG, Heykoop M, Malysheva E, Malysheva V, Martín MP, Morozova OV, Noisripoom W, Overton BE, Rea AE, Sewall BJ, Smith ME, et al. 2019. Fungal Planet description sheets: 951–1041. Persoonia : Molecular Phylogeny and Evolution of Fungi 43:223–425. doi:10.3767%2Fpersoonia.2019.43.06 |  |
| ***kunmudlutsa*** | Henkel TW, Aime MC, Uehling JK, Smith ME (2011) New species and distribution records for *Clavulina* (Cantharellales, Basidiomycota) from the Guiana Shield. Mycologia 103(4):883–894. doi:10.3852/10-355 |  |
| *lilaceorosea* | Huang XX, Chen YX, Lin ZJ, Liang YS, Qiu LH (2023) Three new *Clavulina* species from Baiyun Mountain, Guangzhou, China. Mycological Progress 22(85). https://doi.org/10.1007/s11557-023-01929-x |  |
| *lilliputiana* | Trappe JM, Castellano MA (2007) *Clavulina lillputiana*, a diminutive new species from Tasmania. Australasian Mycologist 25(3):87–89. |  |
| *livida* | He G, Chen S-L, Yan S-Z. 2016. Morphological and molecular evidence for a new species in *Clavulina* from southwestern China. Mycoscience 57(4):255–263. doi:10.1016/j.myc.2016.03.002 |  |
| *mahiscolorata* | de Meiras-Ottoni A, Gibertoni TB (2023) Clavarioid fungi from Brazil: novelties in *Clavulina* (Cantharellales). Mycological Progress 22:25. doi:10.1007/s11557-023-01873-w |  |
| *minor* | Huang XX, Chen YX, Lin ZJ, Liang YS, Qiu LH (2023) Three new *Clavulina* species from Baiyun Mountain, Guangzhou, China. Mycological Progress 22(85). https://doi.org/10.1007/s11557-023-01929-x |  |
| ***monodiminutiva*** | Henkel TW, Meszaros R, Aime MC, Kennedy A (2005b) New *Clavulina* species from the Pakaraima mountains of Guyana. Mycological Progress 4:343–350. doi:10.1007/s11557-006-0140-6 |  |
| ***nigricans*** | Thacker JR, Henkel TW (2004) New species of *Clavulina* from Guyana. Mycologia 96(3):650–657. doi:10.1080/15572536.2005.11832961 |  |
| *ossea* | Tibpromma S, Hyde KD, Jeewon R, Maharachchikumbura SSN, Liu J-K, Bhat DJ, Jones EBG, McKenzie EHC, Camporesi E, Bulgakov TS, Doilom M, Azevedo Santiago ALCM de, Das K, Manimohan P, Gibertoni TB, Lim YW, Ekanayaka AH, Thongbai B, Lee HB, Yang J-B, Kirk PM, Sysouphanthong P, Singh SK, Boonmee S, Dong W, Raj KNA, Latha KPD, Phookamsak R, Phukhamsakda C, et al. 2017. Fungal diversity notes 491–602: taxonomic and phylogenetic contributions to fungal taxa. Fungal Diversity 83:1–261. doi:10.1007/s13225-017-0378-0 |  |
| ***pakaraimensis*** | Uehling JK, Henkel TW, Aime MC, Vilgalys R, Smith ME (2012a) New species and distribution records for *Clavulina* (Cantharellales, Basidiomycota) from the Guiana Shield, with a key to the lowland neotropical taxa. Fungal Biology 116(12):1263–1274. doi:10.1016/j.funbio.2012.09.004 |  |
| *pallidorosea* | Deng PT, Liu XF, Yan J, Zhang P (2024) Update on the taxonomy of *Clavulina* in China II: description of four new species from Hainan Island. Mycological Progress 23:48. doi:10.1007/s11557-024-01988-8 |  |
| *paraincrustata* | Tibpromma S, Hyde KD, Jeewon R, Maharachchikumbura SSN, Liu J-K, Bhat DJ, Jones EBG, McKenzie EHC, Camporesi E, Bulgakov TS, Doilom M, Azevedo Santiago ALCM de, Das K, Manimohan P, Gibertoni TB, Lim YW, Ekanayaka AH, Thongbai B, Lee HB, Yang J-B, Kirk PM, Sysouphanthong P, Singh SK, Boonmee S, Dong W, Raj KNA, Latha KPD, Phookamsak R, Phukhamsakda C, et al. (2017) Fungal diversity notes 491–602: taxonomic and phylogenetic contributions to fungal taxa. Fungal Diversity 83:1–261. doi:10.1007/s13225-017-0378-0 | Synonymized with *C. incrustata* by de Meiras-Ottoni & Gibertoni (2023) |
| *parvispora* | de Meiras-Ottoni A, Gibertoni TB (2023) Clavarioid fungi from Brazil: novelties in *Clavulina* (Cantharellales). Mycological Progress 22:25. doi:10.1007/s11557-023-01873-w |  |
| *perplexa* | Campo E, Franchi P, Marchetti M (2023) *Clavulina* *perplexa*, una nuova specie trovata in Friuli-Venezia Giulia. Rivista Di Micologia 66(1):81–95. |  |
| *purpurascens* | Wu C-L, He Y, Yan J, Zhang P (2019) Two new species of *Clavulina* (Cantharellales) from southwestern China based on morphological and molecular evidence. Mycological Progress 18:1071–1078. doi:10.1007/s11557-019-01506-1 |  |
| ***rosiramea*** | Uehling JK, Henkel TW, Aime MC, Vilgalys R, Smith ME (2012a) New species and distribution records for *Clavulina* (Cantharellales, Basidiomycota) from the Guiana Shield, with a key to the lowland neotropical taxa. Fungal Biology 116(12):1263–1274. doi:10.1016/j.funbio.2012.09.004 |  |
| *sphaeropedunculata* | Yuan H-S, Lu X, Dai Y-C, Hyde KD, Kan Y-H, Kušan I, He S-H, Liu N-G, Sarma VV, Zhao C-L, Cui B-K, Yousaf N, Sun G, Liu S-Y, Wu F, Lin C-G, Dayarathne MC, Gibertoni TB, Conceição LB, Garibay-Orijel R, Villegas-Ríos M, Salas-Lizana R, Wei T-Z, Qiu J-Z, Yu Z-F, Phookamsak R, Zeng M, Paloi S, Bao D-F, et al. (2020) Fungal diversity notes 1277–1386: taxonomic and phylogenetic contributions to fungal taxa. Fungal Diversity 104:1–266. doi:10.1007/s13225-020-00461-7 |  |
| *simplex* | de Meiras-Ottoni A, Gibertoni TB (2023) Clavarioid fungi from Brazil: novelties in *Clavulina* (Cantharellales). Mycological Progress 22:25. doi:10.1007/s11557-023-01873-w |  |
| *spina* | Liu XF, Deng PT, Yan J, Zhang P (2024) Update on the taxonomy of *Clavulina* in China I: description of three new species and one newly recorded species. Mycological Progress 23:45. doi:10.1007/s11557-024-01980-2 |  |
| *studerae* | de Meiras-Ottoni A, Gibertoni TB (2023) Clavarioid fungi from Brazil: novelties in *Clavulina* (Cantharellales). Mycological Progress 22:25. doi:10.1007/s11557-023-01873-w |  |
| ***tepurumenga*** | Henkel TW, Aime MC, Uehling JK, Smith ME (2011) New species and distribution records for Clavulina (Cantharellales, Basidiomycota) from the Guiana Shield. Mycologia 103(4):883–894. doi:10.3852/10-355 |  |
| *terminalis* | de Meiras-Ottoni A, Gibertoni TB (2023) Clavarioid fungi from Brazil: novelties in *Clavulina* (Cantharellales). Mycological Progress 22:25. doi:10.1007/s11557-023-01873-w |  |
| *thindii* | Phookamsak R, Hyde KD, Jeewon R, Bhat DJ, Jones EBG, Maharachchikumbura SSN, Raspé O, Karunarathna SC, Wanasinghe DN, Hongsanan S, Doilom M, Tennakoon DS, Machado AR, Firmino AL, Ghosh A, Karunarathna A, Mešić A, Dutta AK, Thongbai B, Devadatha B, Norphanphoun C, Senwanna C, Wei D, Pem D, Ackah FK, Wang G-N, Jiang H-B, Madrid H, Lee HB, et al. (2019) Fungal diversity notes 929–1035: taxonomic and phylogenetic contributions on genera and species of fungi. Fungal Diversity 95:1–273. doi:10.1007/s13225-019-00421-w |  |
| *tomentosa* | Deng PT, Liu XF, Yan J, Zhang P (2024) Update on the taxonomy of *Clavulina* in China II: description of four new species from Hainan Island. Mycological Progress 23:48. doi:10.1007/s11557-024-01988-8 |  |
| *tuxtlasana* | Pérez-Pazos E, Villegas-Ríos M, Garibay-Orijel R, Salas-Lizana R (2019) Two new species of *Clavulina* and the first record of *Clavulina reae* from temperate *Abies religiosa* forests in central Mexico. Mycological Progress 18:1187–1200. doi:10.1007/s11557-019-01516-z |  |
| *wisoli* | Härkönen M, Niemelä T, Mwasumbi L (2003) Tanzanian Mushrooms Edible, Harmful and Other Fungi. Botanical Museum, Finnish Museum of Natural History, University of Helsinki, Helsinki. |  |
| *yunnanensis* | Liu XF, Deng PT, Yan J, Zhang P (2024) Update on the taxonomy of *Clavulina* in China I: description of three new species and one newly recorded species. Mycological Progress 23:45. doi:10.1007/s11557-024-01980-2 |  |
